# A Roadmap towards Successful Nanocapsule Synthesis via Vesicle Templated RAFT-Based Emulsion Polymerization

**DOI:** 10.3390/polym10070774

**Published:** 2018-07-15

**Authors:** Wendy Rusli, Alexander W. Jackson, Alexander van Herk

**Affiliations:** Institute of Chemical and Engineering Sciences, 1 Pesek Road, Jurong Island 627833, Singapore; wendy_rusli@ices.a-star.edu.sg (W.R.); alexander_jackson@ices.a-star.edu.sg (A.W.J.)

**Keywords:** vesicles, templating polymerization, RAFT, nanocapsules, pro-trusion

## Abstract

Vesicle templated emulsion polymerization is a special form of emulsion polymerization where the polymer is grown from the outside of the vesicle, leading to nanocapsules. Cost effective nanocapsules synthesis is in high demand due to phasing out of older methods for capsule synthesis. Although the first indications of this route being successful were published some 10 years ago, until now a thorough understanding of the parameters controlling the morphologies resulting from the template emulsion polymerization was lacking. Most often a mixture of different morphologies was obtained, ranging from solid particles to pro-trusion structures to nanocapsules. A high yield of nanocapsules was not achieved until now. In this paper, the influence of initial vesicle dispersion, choice of the Reversible Addition-Fragmentation chain Transfer (RAFT) species and oligomer, monomer and crosslinker have been investigated. It turns out that good initial vesicle dispersion, molecular control of the RAFT process, a not too hydrophobic monomer and some crosslinking is needed to result in high yield of nanocapsules. In previous work, the level of RAFT control was often suboptimal and not properly verified and although nanocapsules were shown, other morphologies were also present. We now believe we have a full understanding of vesicle templated nanocapsules synthesis, relevant to many applications.

## 1. Introduction

Emulsion polymerization in general is seen as a process where polymerization takes place in micelles and monomer is supplied by monomer droplets to the growing polymer chains within those micelles. The process typically results in solid latex particles in the range between 50 and 200 nm [1]. In the late 1990s the idea came about in the group of Prof. Anton German to utilize vesicles instead of micelles as the locus of polymerization in an emulsion polymerization, hopefully resulting in hollow nanocapsules instead of solid latex particles. In this context we refer to nanocapsules as capsules that are below 1000 nm in diameter but are not necessarily below 100 nm. This concept was studied intensely but it was observed that the general outcome of such a vesicle templated emulsion polymerization was a phase separated structure where the polymer formed a bead or multiple beads, connected to the vesicle structure [2] (the so-called parachute morphology or necklace morphology respectively). Attempts to maintain an even distribution of the polymer within the vesicle bilayer through adding crosslinking monomers, varying the vesicle forming surfactant and monomer combinations did lead to changes in morphology but not to nanocapsules [2]. It was only after introducing reactivity of the surfactant molecules to copolymerize with the monomer that nanocapsule structures were observed [3].

In order to make this process cost-effective without a need to synthesize complex reactive surfactant molecules, other approaches were needed. Brian Hawkett developed a method for encapsulation, based on the adsorption of Reversible Addition-Fragmentation chain Transfer (RAFT) moiety containing oligomers that could further chain extend from a surface. This method was first published in 2008 in order to encapsulate inorganic particles like Titanium Dioxide [4]. Later the Eindhoven group utilized this method for the encapsulation of Gibbsite platelets [5] and Cerium Oxide particles [6]. Also natural clay platelets (Montmorillonite) were encapsulated, both with conventional and RAFT approaches [7]. Surprisingly it was shown that not only did the RAFT approach work for successful encapsulation, but also that conventional free radical polymerization could work under certain circumstances [7]. The interplay of kinetics and thermodynamics is paramount in determining the resulting morphology [8]. This was also shown in a parallel approach, utilizing Atom Transfer Radical Polymerization (ATRP) instead of RAFT, which was investigated for Gibbsite encapsulation [9,10,11]. From these studies we learned that the ATRP approach is most likely to result in obtaining equilibrium morphologies, whereas free radical polymerization under slow monomer addition can capture non-equilibrium morphologies. The reason is that in ATRP and RAFT, the initial molecular weight is low and building up slowly whereas in free radical polymerization the initial molecular weight is already high from the start. As a result, forming high molecular weight polymer initially, in combination with a low monomer concentration in the growing particles restricts mobility of the molecules and particles and can capture non-equilibrium morphologies [7,8,10]. Alternatively, crosslinking can also capture non-equilibrium morphologies [11].

In vesicle templated emulsion polymerization, it seems that the typical equilibrium morphology obtained is the parachute morphology [2]. Similarly to the encapsulation of inorganic particles, in this paper we want to see how we can utilize the interplay of kinetics and thermodynamics to obtain nanocapsule through vesicle templated emulsion polymerization (as a non-equilibrium morphology). 

The RAFT oligomer approach was therefore also applied to the vesicle templated emulsion polymerization, with the assumption that the negatively charged RAFT oligomers would adsorb on the outside of the positively charged dimethyldioctadecylammonium bromide (DODAB) vesicles, fortifying the vesicle structure. The RAFT oligomer is then chain extended, resulting in nanocapsules. In comparison to the earlier success in utilizing reactive surfactants [3], this approach is more closely resembling a normal emulsion polymerization in terms of chemicals; regular non-reactive (in this case vesicle forming) surfactants, typical monomers, a transfer agent (in this case a RAFT agent) and a normal initiator. The only extra step is to adsorb the RAFT oligomer onto the preformed vesicles, but after that this vesicle templated emulsion polymerization is very similar to a regular emulsion polymerization. As RAFT agents are now commercially available at large quantities and at a low price, this approach seemed to be a cost-effective platform technology for the large-scale production of nanocapsules. The initial results seemed promising [12,13]. However, the level of molecular and morphological control was not well established at that time. 

The technique of choice to visualize the resulting morphologies is cryo-Transmission Electron Microscopy (cryo-TEM). In cryo-TEM the aqueous dispersion of the resulting particles is frozen rapidly and as such this technique is able to capture even the most delicate structures, including vesicles. There are also some drawbacks to cryo-TEM. For example, the thin ice layer formed in the orifice tends to create some size segregation. Related to that, in most pictures of parachute morphologies the polymer bead is always positioned at the edge of the structure in the 2D projection, the bead is hardly ever seen in the middle of the structure. So, in order to elude the predominant morphology, it is important to identify a large number of particles with their corresponding morphologies. A quantitative study on the morphologies of vesicle templated emulsion polymerizations revealed that there are often several morphologies in a system and it can be risky to selectively only analyze a limited number of particles of certain morphology in a publication [14]. Therefore, we partially doubt some of our own conclusions in the earlier work [12,13]. 

Nanocapsules find their use in many applications like controlled drug release, personal care and consumer care products [15]. Some of the older technologies for preparing nanocapsules are currently phased out due to legislation/toxicity issues and also in several countries polymer microbeads are banned. So the demand for new technologies to prepare nanocapsules in a cost-effective way is growing (in this paper the nanocapsules are typically between 130 and 400 nm).

**Figure 1:**
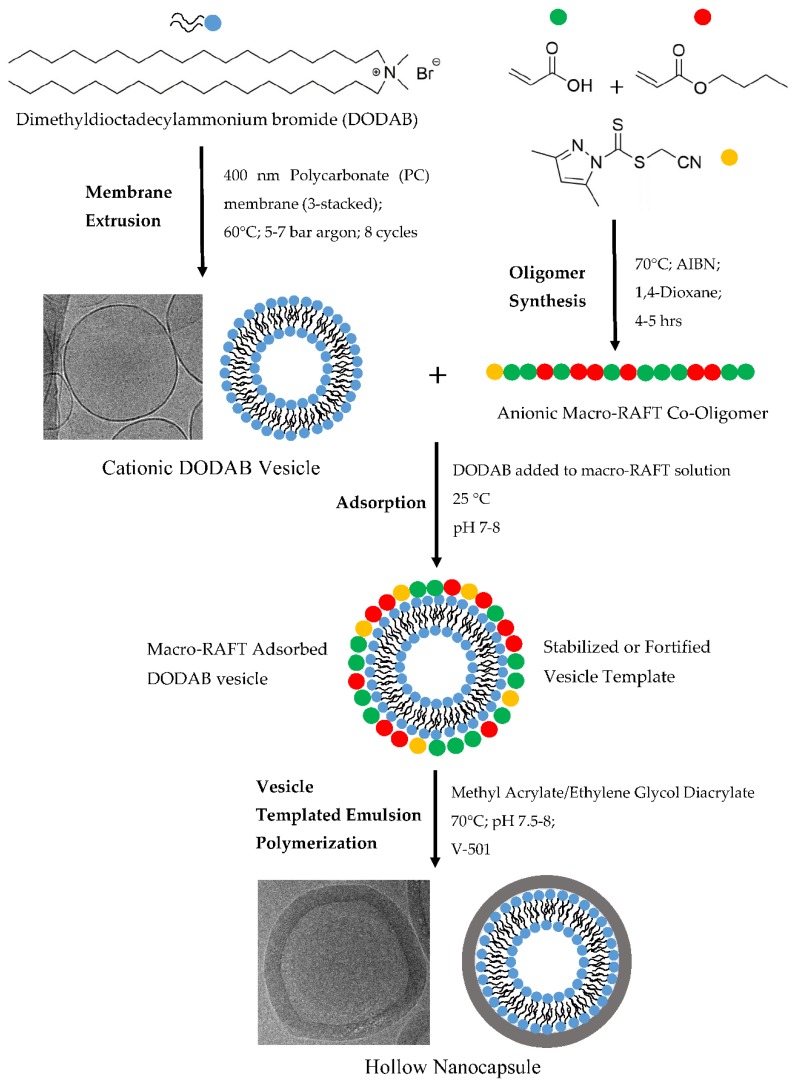
Nanocapsule synthesis via vesicle templated RAFT-based emulsion polymerization.

In this paper, we would like to present a thorough and final study on the parameters that control the morphology outcome of vesicle templated emulsion polymerizations utilizing the RAFT oligomer approach. Figure 1 shows the nanocapsules synthesis scheme.

## 2. Materials and Methods 

### 2.1. Materials

Dimethyldioctadecylammonium bromide (DODAB, Sigma Aldrich, St. Louis, MO, USA, ≥98%) was used as received. Butyl acrylate (BA, ≥99%, Sigma Aldrich), methyl acrylate (MA, 99%, Sigma Aldrich), methyl methacrylate (MMA, 99%, Sigma Aldrich), ethylene glycol diacrylate (EGDA, 95%, Polysciences, Warrington, PA, USA) and ethylene glycol dimethacrylate (EGDMA, 98%, Sigma Aldrich) were purified by passing through a column of basic alumina (Sigma Aldrich) in argon atmosphere. Acrylic acid (AA, 99%, Sigma Aldrich), 1,3,5-trioxane (≥99%, Sigma Aldrich), 1-dodecanethiol (≥98%, Sigma Aldrich), *N*,*N*-Dimethylformamide (DMF, anhydrous 99.8%, Sigma Aldrich), tetrahydrofuran (THF, anhydrous, ≥99.9%, Sigma Aldrich) and 1,4-dioxane (99.8%, Sigma Aldrich) were used as received. 

Two RAFT agents cyanomethyl (3,5-dimethyl-1*H*-pyrazole)-carbodithioate (BM1481, >95%) and dibenzyl trithiocarbonate (DBTTC, BM1361, >95%) were obtained from Boron Molecular (Noble Park, Australia) and were used as received. 

Two free radical initiators for polymerization: 2,2′-azobisisobutyronitrile (AIBN, Wako Chemical, Osaka, Japan) and 4,4′-azobis (4-cyanovaleric acid) (V-501, Wako Chemical) were used as received. 

De-ionized water (DI water, AquaMaX^TM^ Basic 321 System, Anyang, South Korea) and sodium chloride (NaCl, VWR, Radnor, PA, USA) were used for the vesicle preparation. The water was tapped into a glass bottle directly from the purification unit only prior to vesicle hydration.

### 2.2. Preparation of Unilamellar DODAB Vesicles

DODAB vesicles were prepared as a 10 mM (0.631 wt%) solution in 0.5 mM NaCl_(aq)_ [16]. Typically, a 100 g mixture was prepared, stirred and heated in 65 °C oil bath for at least 18 h prior to extrusion. The extrusion was performed on Millipore (Burlington, MA, USA) SS filter holder (47 mm) by forcing the vesicles through 3 stacked 400 nm polycarbonate (PC) membranes (Millipore, HTTP04700) for 8 cycles at 60 °C, 5–7 bar argon. The extruded vesicles were then allowed to cool to room temperature. Dynamic Light Scattering (DLS) was used the determine the size of the vesicles.

### 2.3. Preparation of Macro-RAFT Co-Oligomer

Two random macro-RAFT oligomers were prepared by polymerizing a mixture of butyl acrylate and acrylic acid in the presence of RAFT agent and initiator. The synthesis procedure was as follows, the weighed amount of RAFT agent ((BM1481, 1.5 g, 7.1 mmol, 1 eq) or (BM1361, 1.5 g, 5.2 mmol, 1 eq)) was transferred into a Schlenk flask containing, on average, 6 units of butyl acrylate (BA) and 9 units of acrylic acid (AA), hence referred as BM1481 (BA_6_-*co*-AA_9_), also known as **Oligo 1** or BM1361 (BA_6_-*co*-AA_9_), also known as **Oligo 2A**. The initiator (AIBN, 0.1 eq) was then added followed by 1,4-dioxane (10.3 g, 117 mmol) as the reaction medium. It was then stirred magnetically to obtain a clear bright-yellow liquid. The flask was then sealed with rubber septa and subjected to 3 freeze-pump-thaw cycles. The polymerization was started by heating the flask to 70 °C for 4-5 h under argon atmosphere at constant stirring speed. The polymerization was stopped by quenching in liquid nitrogen and the product was precipitated out from hexane to obtain a yellow fluffy powder. Matrix Assisted Laser Desorption/Ionization Time of Flight (MALDI-ToF) Mass Spectrometer (MS), Gel Permeation Chromatography (GPC) in THF and Nuclear Magnetic Resonance (NMR) spectroscopy were used to characterize the oligomer.

### 2.4. Preparation of RAFT-Less Co-Oligomer

Co-oligomer without RAFT moiety was synthesized via free radical polymerization. The procedures were as follows. Butyl acrylate (2.87 g, 22 mmol), acrylic acid (2.74 g, 38 mmol), 1-dodecanethiol (0.8 g, 4 mmol), AIBN (4.3 mg, 26 µmol) and 1,4-dioxane (5 mL) were mixed and transferred in a 10 mL Schlenk flask. The solution was subjected to 3 freeze-pump-thaw cycles, back-filled with argon and heated to 70 °C. The polymerization was terminated after 30 min by quenching in liquid nitrogen. The product was precipitated from hexane and referred as **Oligo 3**. Characterization of the co-oligomer was done with GPC (THF) and NMR.

### 2.5. Solution Polymerization of Macro-RAFT Co-Oligomer

A typical solution polymerization was performed as follows. The macro-RAFT co-oligomer (**Oligo 1**, 0.48 g, 0.3 mmol, 1 eq), methyl acrylate (2.58 g, 30 mmol, 100 eq), V-501 (8.4 mg, 30 µmol, 0.1 eq) and DMF (8.77 g, 120 mmol, 4:1 by mol with respect to monomer) were transferred into a 25 mL Schlenk tube. The solution was degassed via 3 freeze-pump-thaw cycles, back-filled with N_2_ and heated to 70 °C. At regular time intervals small aliquots were extracted for ^1^H-NMR (CDCl_3_) spectroscopy and GPC (DMF) analysis in order to determine monomer conversion and molecular weight evolution, respectively.

### 2.6. Adsorption of Macro-RAFT Co-Oligomer to the Vesicle Surface

Adsorption of anionic macro-RAFT co-oligomer to cationic DODAB vesicle was done by feeding the cationic DODAB vesicle to the macro-RAFT solution at room temperature. To do this, an aqueous stock solution of 10 mM macro-RAFT was prepared by dissolving the macro-RAFT with dilute NaOH_(aq)_ to give a solution with pH 7. Different concentrations of macro-RAFT co-oligomer were then prepared in 20–25 glass vials. Each of the vials was first filled with different amount of macro-RAFT solution (mL) and was then diluted with water to make a solution of 1 mL. Under stirring, an equal volume (1 mL) of extruded DODAB vesicles (10 mM) was then fed to each of the vials at 1 mL/min. Size and zeta potential measurement (DLS) were then performed on every vials. The adsorption procedure has been reported elsewhere [12,14].

### 2.7. DODAB Vesicle Polymerization (Transcriptive Synthesis)

Vesicle polymerization was conducted in a 100–250 mL jacketed glass reactor equipped with 4-morton indents and 3-bladed impeller (45°). The amount of macro-RAFT and DODAB for polymerization was determined by the result of the adsorption study (Section 2.6). Typically, the required amount of macro-RAFT solution (10 mM, pH 7) and water was charged into the reactor, DODAB (10 mM, pH ~ 7–8) was then added dropwise using a syringe pump (2 mL/min) at room temperature under constant stirring speed (300–400 rpm) to give a slightly milky-yellowish translucent mixture with pH of 7.5–8. The content was then purged with argon for 30–60 min. The reactor was then heated to 70 °C. Monomer or monomer mixtures (pre-purged with argon) were then starved-fed (1 mL/h) into the reactor using a syringe pump followed by injection of initiator solution (V-501, pH ~ 7, pre-purged with argon). The amount of monomer(s) fed was 1 g for every 100 mL mixture of DODAB and macro-RAFT co-oligomer solution. At the end of monomer feeding, the stirring speed was lowered to 150–200 rpm and the content was allowed to react overnight. The polymerization was stopped by cooling the reactor to room temperature and allowing air to diffuse into the reactor. Unreacted monomer was removed by purging with argon. The morphology of the resulting nanocapsules was analyzed with cryo-TEM and DLS was used to determine the size and distribution of the particles.

### 2.8. Vitrification and Cryo-Transmission Electron Microscope (Cryo-TEM)

Vitrification of the vesicles and nanocapsules samples was carried out using a vitrification robot (FEI Vitrobot Mark IV, Hillsboro, OR, USA). All samples were prepared at room temperature and 100% humidity with blotting time of 2 s and blot force of 1. The sample (5 μL) was applied onto a grid (Quantifoil, R2/2, Holey carbon film; freshly glow-discharged prior to use at 20 mA for 60 s) without dilution. Excess of sample was blotted away with filter paper to leave a thin film on the grid before being vitrified in liquid ethane. Cryo-TEM measurements were performed on FEI Titan Krios equipped with automated sample loader and a Field Emission Gun (FEG) operating at 300 kV. Images were recorded with Falcon II camera (4 × 4) with magnification of 29,000 and pixel size of 2.873 Å.

### 2.9. Dynamic Light Scattering (DLS)

Size and polydispersity (PDI) of the vesicles were measured using ZetaSizer Nano ZS-DLS (Malvern Panalytical, Malvern, UK) in a standard 12 mm polystyrene (PS) cuvette. The sample was measured at 25 °C without any dilution at 173° backscatter. Measurement position and attenuation was optimized by the instrument. A mean size (*z*-*ave*) and polydispersity index (PDI) was determined in a standard cumulant analysis of the software whereas a general purpose (normal distribution) algorithm from the software was used for size distribution analysis.

### 2.10. Matrix Assisted Laser Desorption/Ionization Time of Flight (MALDI-ToF) Mass Spectrometer (MS)

MALDI-ToF MS (Bruker, Bremen, Germany) analysis of the macro-RAFT co-oligomer was carried out using Bruker Autoflex Speed System using *trans*-2-[3-(4-*tert*-Butylphenyl)-2-methyl-2-propenylidene] malononitrite (DCTB) matrix in linear negative mode of ionization. The matrix was dissolved in THF at 10 mg/mL. The sample were prepared at 2 mg/mL in THF and mixed with the matrix in 10:1 ratio by volume. Each mass spectrum was collected with *app.* 5000 laser shots in linear negative mode. The molecular weight of oligomers was calculated using the Flex analysis™ (Bruker) and Polymerix software™ (Sierra Analytics, Modesto, CA, USA).

## 3. Results and Discussion

### 3.1. Preparation and Characterization of DODAB Vesicle as Template

**Figure 2:**
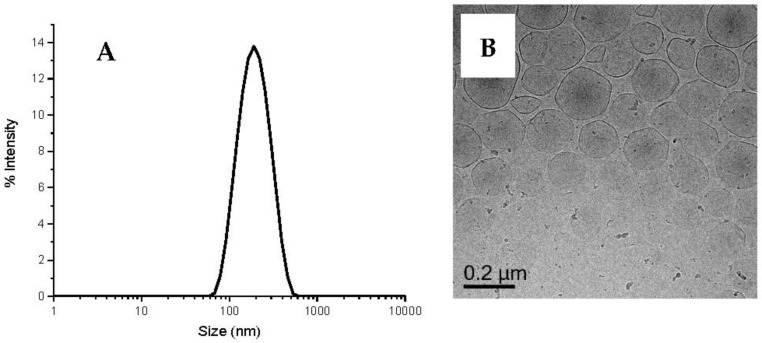
Size distribution (**A**) and cryo-TEM micrograph (**B**) of DODAB vesicles (10 mM) prepared in 0.5 mM NaCl_(aq)_.

DODAB vesicles (10 mM) were prepared in 0.5 mM NaCl_(aq)_ and then hydrated at 60 °C for 18 h prior to extrusion through a 400 nm PC membrane [16]. The 10 mM DODAB is used because it is the critical concentration for DODAB [17]. The extrusion produces vesicles with sizes of several hundred nanometers in a short time period with high recovery. The size (*z-ave*) of the vesicle obtained is 168.6 nm ± 0.4 with polydispersity index (PDI) of 0.18. Figure 2A shows the size distribution of the extruded DODAB vesicles. Figure 2B shows the structure and morphology of DODAB vesicles obtained from cryo-TEM. DODAB appears as a homogeneous quasi-spherical unilamellar vesicle dispersion with size around 170 nm, in agreement with DLS.

### 3.2. Synthesis of Macro-RAFT Co-Oligomer for Vesicle Polymerization

**Figure 3:**
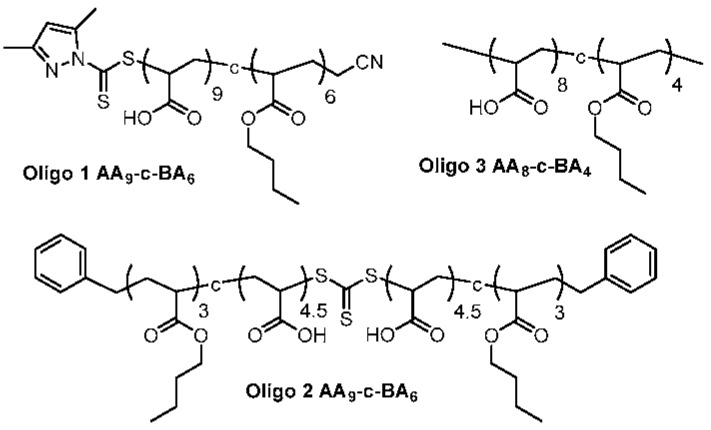
Chemical structures of **Oligo 1** [RAFT1481 (BA_6_-*co*-AA_9_)], **Oligo 2** [RAFT1361 (BA_6_-*co*-AA_9_)] and **Oligo 3** (BA_4_-*co*-AA_8_).

Two macro-RAFT co-oligomers were synthesized in 1,4-dioxane i.e., BM1481 (BA_6_-*co*-AA_9_), also known as **Oligo 1** and BM1361 (BA_6_-*co*-AA_9_) also known as **Oligo 2A** (Figure 3). The effect of solvent on RAFT polymerization was also investigated by synthesizing BM1361 (BA_6_-*co*-AA_9_) in 3 other solvents i.e., isopropanol (**Oligo 2B**), ethanol (**Oligo 2C**) and bulk (**Oligo 2D**). Additionally, we made another oligomer (**Oligo 3**, Figure 3) without RAFT moiety via free radical polymerization in the presence of 1-dodecanethiol, also in 1,4-dioxane as solvent.

**Figure 4:**
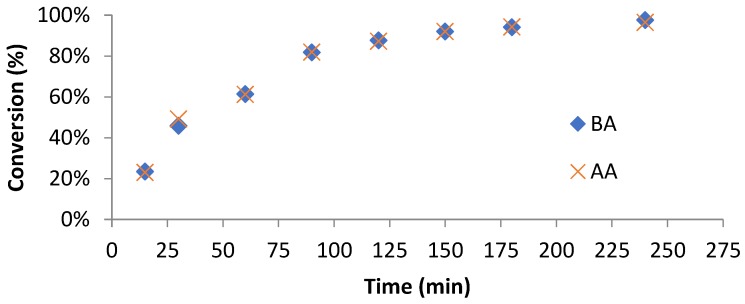
Conversion of butyl acrylate (BA) and acrylic acid (AA) during **Oligo 1** [BM1481 (BA_6_-*co*-AA_9_)] synthesis. Co-polymerization was carried out at 70 °C in 1,4-dioxane (52 wt%) using AIBN as initiator and 1,3,5-trioxane as internal standard for NMR measurement.

**Figure 5:**
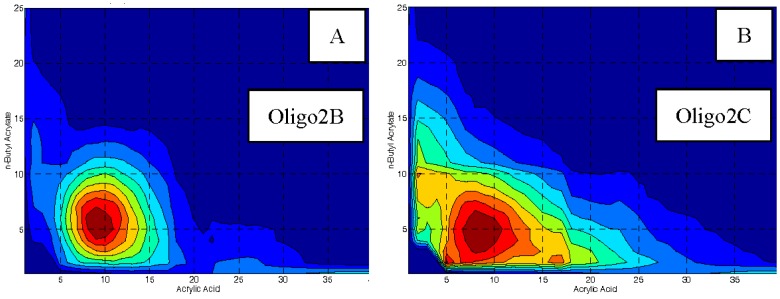
Composition of **Oligo 2** synthesized in isopropanol (**A**) and ethanol (**B**) from MALDI-ToF MS analysis showing the number of acrylic acid units (x-axis) and butyl acrylate units (y-axis).

Synthesis of macro-RAFT is straight forward and well described by Brian Hawkett from Sydney University [4]. Our study is in agreement with his result in which the conversion (NMR, Figure 4) of both BA and AA was almost identical. This means that the macro-RAFT co-oligomer should have a random distribution of hydrophobic and hydrophilic units to prevent the undesirable formation of micelles in aqueous system. Analysis of the three co-oligomers can be seen in Table 1. GPC analysis shows that all macro-RAFT co-oligomers have narrow molecular weight distribution with PDI of 1.3. However, MALDI-ToF analysis shows that even though the average PDI can be relatively narrow; the chemical composition distribution of macro-RAFT co-oligomer can be still broad as can be seen in the fingerprint analysis of **Oligo 2B** and **2C**, synthesized in isopropanol and ethanol as solvent, respectively (Figure 5A,B). It is also possible to synthesize the macro-RAFT co-oligomer without using any solvents, by simply use the monomer as the reaction medium, the fingerprint of the macro-RAFT co-oligomer produced in bulk is shown in Figure S1. Therefore it can be concluded that the chemical composition and molecular weight distributions contain a variety of oligomers and at the extremities of these distributions the oligomers might have somewhat different adsorption characteristics and behavior in aqueous solution.

In light of the view that the PDI obtained from GPC for **Oligo 1** and **Oligo 2A** (synthesized in 1,4-dioxane) is the lowest at 1.26 and 1.31, respectively and for direct comparison with previous work by Ali et al. [12,13], both are utilized for adsorption and vesicle polymerization in our work.

Synthesis of **Oligo 3** via free radical polymerization in the presence of 1-dodecanethiol results in an oligomer that is very close in molecular weight and chemical composition to the macro-RAFT co-oligomer. An oligomer with a similar composition to macro-RAFT is obviously more difficult to obtain via free radical polymerization. With NMR, the composition of the oligomer is established as BA_4_-*co*-AA_8_. The molecular weight of the oligomer is 1200 g/mol (GPC, Table 1). **Oligo 3** should behave as anionic polyelectrolytes in the aqueous system to be utilized for vesicles polymerization.

### 3.3. Chain Extension of Macro-RAFT Co-Oligomer in Solution

In applying the RAFT technology in heterogenous systems, the distribution of the monomers and oligomers is non-homogenous. Also, sometimes the analysis of the formed polymers is complicated by the presence of the surfactants and side reactions. Therefore the level of control was first of all established in solution polymerization.

**Figure 6:**
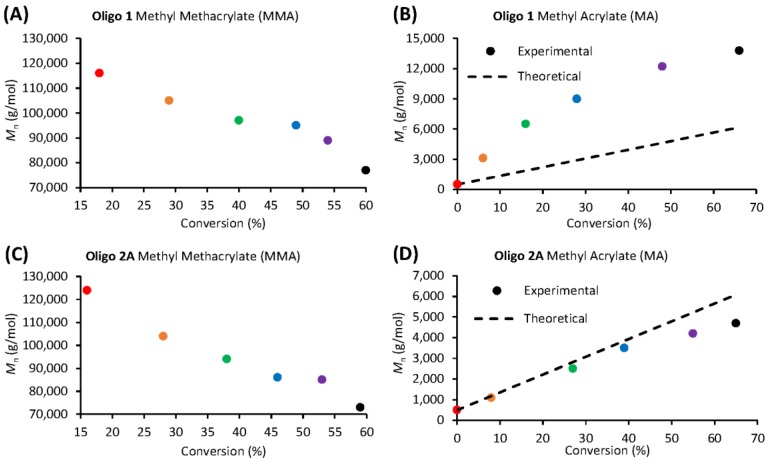
Number average molecular weight vs conversion plots for RAFT oligomer solution polymerizations in DMF (**A**) **Oligo 1** + MMA, (**B**) **Oligo 1** + MA, (**C**) **Oligo 2A** + MMA and (**D**) **Oligo 2A** + MA.

**Figure 7:**
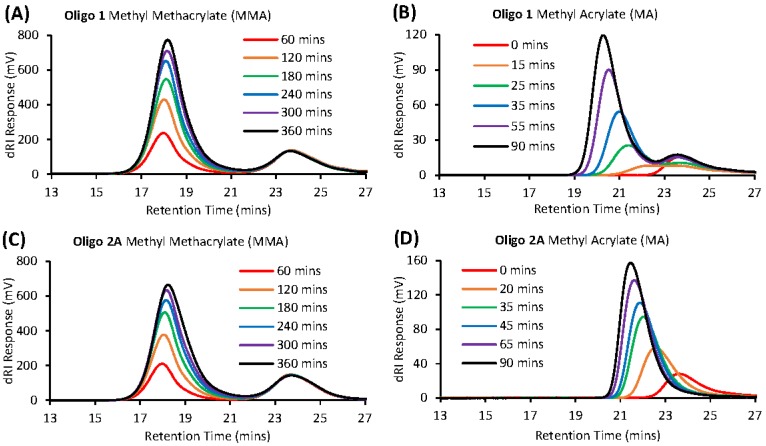
Gel permeation chromatography (in THF) traces for macro-RAFT co-oligomer solution polymerizations in DMF (**A**) **Oligo 1** + MMA, (**B**) **Oligo 1** + MA, (**C**) **Oligo 2A** + MMA and (**D**) **Oligo 2A** + MA.

Each acrylate-based macro-RAFT co-oligomer (**Oligo 1** and **Oligo 2A**) were screened for their ability to control the polymerization of either methyl acrylate (MA) or methyl methacrylate (MMA) via solution polymerization in DMF. We clearly observed that both of the acrylate-based oligomers (**Oligo 1** and **Oligo 2A**) are unable to control the polymerization of MMA. Figure 6A,C show that the molecular weight decreases with increasing monomer conversion, indicative of an uncontrolled free radical polymerization. The molecular weight of **Oligo 1** and **Oligo 2A** increases slowly when polymerized with MA (Figure 6B,D). This conclusion is further backed up by the lack of oligomer consumption, Figure 7A,C display the respective oligomer peak at 24 min, this peak does not deplete or increase in molecular weight during the polymerization. On the other hand, each oligomer (**Oligo 1** and **Oligo 2A**) were able to control the polymerization of MA, albeit to different extents.

**Oligo 1** displays an increase in molecular weight with increasing monomer conversion (Figure 6B) which is a good indication of a controlled/living radical polymerization. However, we observe a significant deviation from the theoretical molecular weight evolution. Figure 7B displays a small proportion of unreacted oligomer (peak at 24 min) even at 90 min of reaction time. This unreacted oligomer shifts the ratio of monomer to (active) oligomer and results in higher molecular weights than expected. Conversely, **Oligo 2A** was excellent at controlling the polymerization of MA. Figure 6D shows that the molecular weight increase with increasing monomer conversion and this molecular weight evolution tracks very close to the theoretical molecular weight evolution. In Figure 7D we can see that all of the oligomer is consumed and chain-extends during the polymerization.

### 3.4. Adsorption of Macro-RAFT Co-Oligomer on the DODAB Vesicle Surface

**Figure 8:**
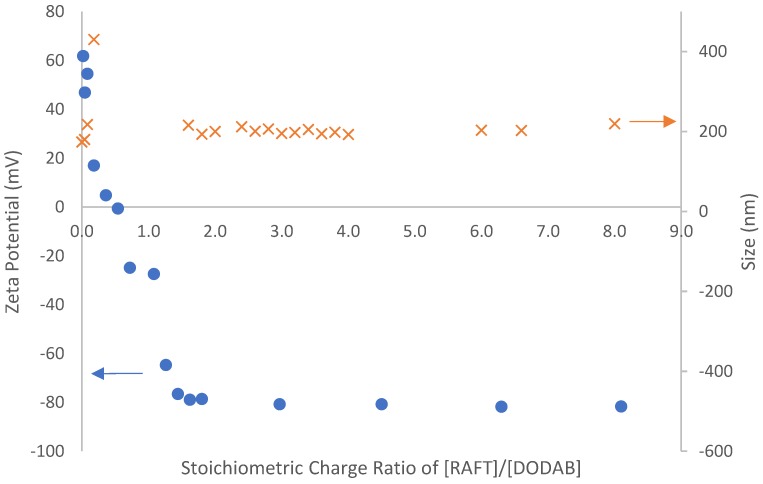
Adsorption of **Oligo 2A** [BM1361 (BA_6_-*co*-AA_9_)] to DODAB vesicle as a function of stoichiometric charge ratio parameter.

To facilitate polymerization on the vesicle surface (transcriptive synthesis), adsorption of anionic macro-RAFT co-oligomer was performed on the cationic DODAB vesicle. It is done by feeding the vesicle (10 mM) to vials containing different concentrations of macro-RAFT. The process is purely electrostatically driven and it results in charge-reversal of the vesicles. Based on the previous work in Eindhoven University of Technology [12,13], the adsorption is based on the assumptions that only the surfactant in the outer layer of the vesicles is interacting with the oligomer and the number of surfactant molecules in both outer an inner layer is the same. This implies that a point of zero charge corresponds to a charge ratio parameter of 0.5 (from the equation below). The stoichiometric charge ratio parameter (ξ) is used to quantify the composition of the mixture during adsorption and is expressed by:

$$\xi =N_{\mathrm{acid}}\frac{\left[\mathrm{RAFT}\right]}{\left[\mathrm{DODAB}\right]}$$

where N_acid_ is the number of acrylic acid unit in the oligomer, [RAFT]/[DODAB] is the molar ratio of RAFT oligomer and DODAB vesicles. Figure 8 shows the adsorption profile of **Oligo 2A** [BM1361 (BA_6_-*co*-AA_9_)] to DODAB vesicles.

It can be clearly seen from Figure 8 that the isoelectric point of DODAB vesicles falls at a charge ratio of 0.5 which is in agreement with the assumption that only the surfactant on the surface interacts with the co-oligomer. At this point, a partial loss of colloidal stability occurs in which the vesicles are loosely coagulated. As the amount of **Oligo 2A** is increased, there is enough co-oligomer on both the vesicle surface and the aqueous phase to result in charge inversion of the vesicles (anionic DODAB) and regaining colloidal stability. The first point of both charge reversal and colloidal stability is achieved at stoichiometric charge ratio of 1.6, but to ensure that no aggregation occurs, additional co-oligomer is used for the actual vesicle polymerizations. 

### 3.5. Vesicle Templated Polymerization (Trancriptive Synthesis)

Based on the result of the adsorption study, vesicle templated nanocapsules are synthesized via starved feed emulsion polymerization. With this method, monomer build up and droplet formation in the system can be prevented. Otherwise, monomer can diffuse in the bilayer of the vesicle during polymerization that will result in parachute or necklace morphology as described by Jung et al. [2,3,18,19]. Additionally, excess monomer in the system might also compete with the macro-RAFT co-oligomer to give polymer particles as a result of secondary nucleation [12].

The process is started by the adsorption of macro-RAFT co-oligomer at a certain stoichiometric charge ratio, followed by chain extension of the macro-RAFT via additional monomer and initiator. The polymerization recipe can be seen in Table 2. The charge ratio parameter is kept at higher ratio to ensure that there are enough macro-RAFT co-oligomers in the aqueous phase to provide stabilization of the resulting nanocapsules during and after polymerization.

In order to be sure that extruded vesicles and macro-RAFT co-oligomer chain extension is the only combination that results in nanocapsules, we performed several blank experiments (Exp 1–5) followed by investigating different monomers (with and without crosslinker). Table 3 summarizes all the vesicle template polymerizations using **Oligo 1**. The morphologies of the particles obtained in all polymerizations are investigated using cryo-TEM.

#### 3.5.1. Polymerization of **Oligo 1** with DODAB Vesicle (Unextruded) (Exp 1)

One of the best way to obtain unilamellar vesicles with well-controlled size and geometry is via the extrusion method [20]. However, unextruded DODAB is known to exist in multiple morphologies e.g., lamella, small and giant vesicles, multilayer structures etc. and exists in a very broad size distribution (DLS) [21,22]. The adsorption of macro-RAFT co-oligomer to unextruded DODAB vesicle is done by adding the unextruded vesicle to **Oligo 1** solution (pH 7, 10 mM) and it results in major coagulation. This means that without homogenous unilamellar vesicles, there is less surface area than the extruded vesicles for the adsorption of the macro-RAFT and in complex environment of various morphologies and sizes, the colloidal stability is disrupted. Polymerization simply cannot be done in such a system.

#### 3.5.2. Polymerization of MA/EGDA with **Oligo 1** without DODAB Vesicle (Exp 2)

**Figure 9:**
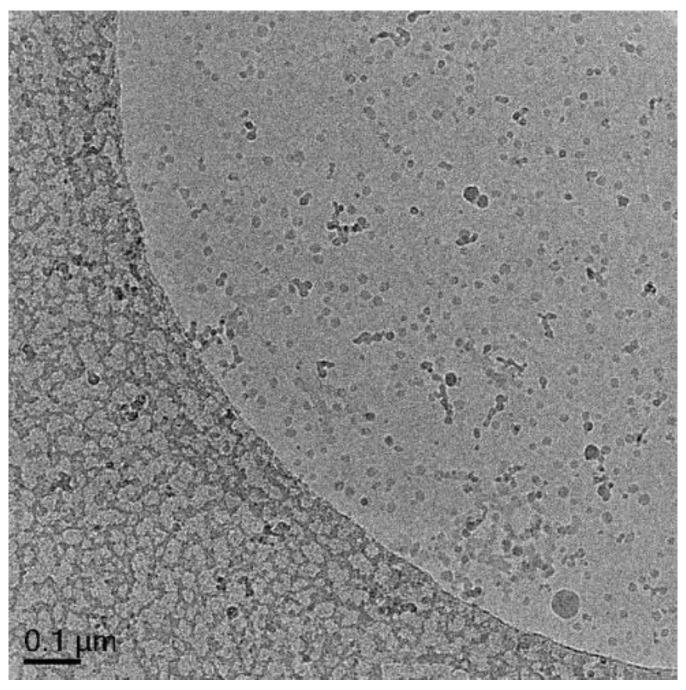
Cryo-TEM micrograph of Exp 2, polymerization of MA/EGDA with **Oligo 1** without DODAB vesicle. Additional images in Figure S2.

The characteristic of macro-RAFT co-oligomer polymerization in solution was investigated by performing a polymerization in aqueous solution without the vesicles. The result from cryo-TEM (Figure 9, Figure S2) shows the presence of small solid particles with size of 10–20 nm. This means that the macro-RAFT co-oligomer has the ability to form nanoparticles in water. Formation of micelles cannot be excluded. A polymer chain with one hydrophilic unit and 4–5 units of hydrophobic unit is enough to behave like micelles in solution. The MALDI-ToF analysis (Figure 5) shows a variety of oligomers is present. Additionally, this micelle-like properties can also develop during the growth of a chain of macro-RAFT co-oligomer, similar to polymerization induced self-assembly (PISA) i.e., the macro-RAFT co-oligomer gets more hydrophobic as the chain grows and gets surface active [23,24,25,26]. This result confirms that dissolved macro-RAFT co-oligomer is not entirely inert in solution, but the polymerization does not result in nanocapsules. In many successful nanocapsule formation reactions, some of these small particles are still present in the background.

#### 3.5.3. Polymerization of MA/EGDA with DODAB Vesicle without **Oligo 1** (Exp 3)

Polymerization of MA/EGDA with unilamellar DODAB vesicles without macro-RAFT co-oligomer was also conducted by starved feeding of monomer. It is important to determine if synthesis of nanocapsules via vesicle templated polymerization (transcriptive synthesis) can be done without the RAFT polymerization technique. We observed unstable dispersions upon polymerization, in which coagulation and precipitation of the vesicles (white solids) occurred. The presence of anionic macro-RAFT co-oligomer is clearly important in stabilizing the vesicles (fortification) and nanocapsules upon polymerization. Without the macro-RAFT and coupled with the formation of solid particles in the aqueous phase, the vesicle structures disintegrated, resulting in major coagulation. We aim to produce stable nanocapsules dispersion and due to this stability issue in Exp 3, we did not record any cryo-TEM image. In a previous study by Jung et al. [18,19], a parachute morphology was obtained, but the monomer (styrene) was stirred in DODAB dispersion for 48 h to allow it to diffuse into the vesicles bilayer prior to polymerization (morphosynthesis/polymerization in the bilayer). We believe that our starved-feed conditions minimize the formation of this morphology.

#### 3.5.4. Polymerization of MA/EGDA with **Oligo 3** without DODAB Vesicle (Exp 4)

**Figure 10:**
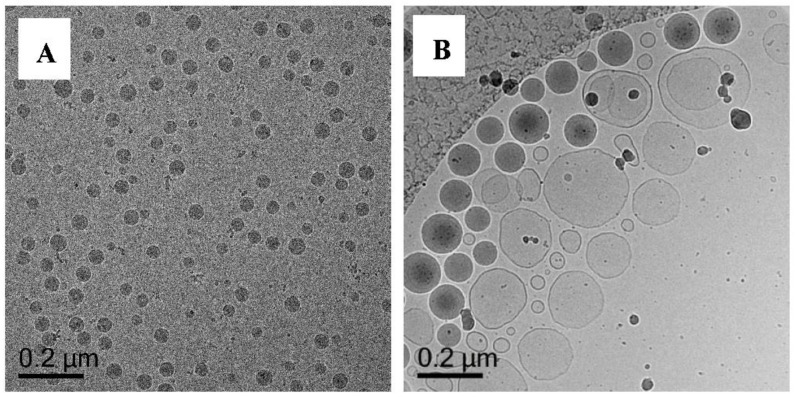
Cryo-TEM micrographs of Exp 4 (**A**), polymerization of **Oligo 3** without DODAB vesicle and Exp 5 (**B**), polymerization of **Oligo 3** with DODAB vesicle. Both polymerized with MA/EGDA as the monomer. Additional images in Figure S3 (Exp 4) and Figure S4 (Exp 5).

We extend our blank study on the synthesis of nanocapsules via fortified vesicle templating by using a RAFT-less co-oligomer (**Oligo 3**, BA_6_-*co*-AA_9_) that we synthesized using free-radical solution polymerization in 1,4-dioxane in the presence of 1-dodecanethiol. The molecular weight (GPC) of the co-oligomer (**Oligo 3**) obtained is 1200 g/mol (Table 1). Using only **Oligo 3** produces small solid particles (30–70 nm) (Figure 10A, Figure S3) when polymerized without DODAB vesicles under starved-feed condition (Exp 4). This is probably the result of precipitation polymerization in which the polymer is formed in the water phase and stabilized by **Oligo 3** [27]. In comparison to Exp 2, this polymerization leads to higher molecular weight in the co-oligomer of the formed polymer and less particles and this probably might be the reason for the larger solid particles [27].

#### 3.5.5. Polymerization of MA/EGDA with **Oligo 3** and DODAB Vesicle (Exp 5)

Prior to polymerization, adsorption of **Oligo 3** on the DODAB vesicle surface is performed also by adding DODAB vesicle to **Oligo 3** solution as described in Table 2. When these RAFT-less oligomer-fortified DODAB vesicles were polymerized, a good stable dispersion is obtained, but cryo-TEM analysis (Figure 10B, Figure S4) shows the presence of two populations of particles i.e., the vesicle and solid particles (100–150 nm). Without RAFT moiety, **Oligo 3** cannot chain-extend on the vesicle surface and thus cannot form nanocapsules and mainly functions in stabilizing the vesicles. The big solid particles in this case probably are the result of secondary nucleation of the monomer in the water phase and the resulting latex particles are stabilized by the free **Oligo 3** in solution. In Exp 3, there is no oligomer utilized and no colloidal stability is obtained after polymerization whereas in Exp 5, colloidal stability is achieved in the presence of anionic **Oligo 3**. Comparing Exp 3 and Exp 5 further strengthen the importance of having free anionic co-oligomer in the aqueous phase to help stabilize the formed particles.

#### 3.5.6. Polymerization of **Oligo 1** with DODAB Vesicle (Exp 6–15)

With the results obtained from “blank experiments” above (Exp 1–5), it can be concluded that successful synthesis of nanocapsules through vesicle templating depends on several conditions below:

- DODAB vesicles as templates must be homogenous in size and exist as unilamellar particles.
- RAFT moiety in the form of macro-RAFT co-oligomer is required for further chain extension of the co-oligomer on the vesicles surface.
- Colloidal stability after adsorption is critical to retain the vesicular structures.
- The choice of monomer is important to achieve molecular control over the polymerization (based on the result from solution polymerization of macro-RAFT co-oligomer).

In the following polymerization, DODAB vesicle is subjected to polymerization using **Oligo 1** by starved feeding various amount of monomers with and without crosslinker. The morphologies of the particles are investigated with cryo-TEM.

##### Polymerization of DODAB-**Oligo 1** with MA (Exp 6) and MMA (Exp 7)

**Figure 11:**
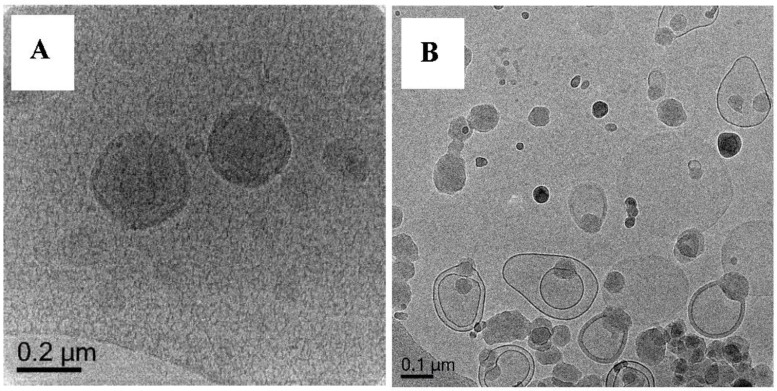
Cryo-TEM micrographs of Exp 6 (**A**), polymerization of (DODAB-**Oligo 1**) with MA and Exp 7 (**B**), polymerization of (DODAB-**Oligo 1**) with MMA. Additional images in Figure S5 (Exp 6) and Figure S6 (Exp 7).

With MA, **Oligo 1** exhibits an excellent control over polymerization whereas poor control is observed for MMA (Section 3.3). Cryo-TEM image of Exp 6 (Figure 11A, Figure S5) shows both capsules and solid morphologies. It is important to note that not many capsule particles can be found during the imaging. This result shows the indication of successful synthesis of nanocapsules i.e., a hollow particle with a clear polymeric shell. On the other hand, polymerization with MMA (Figure 11B, Figure S6) results in solids particles and pro-trusion structures. The pro-trusion morphology [14a, b] is defined as a morphology in which there is a polymer blob with a polymer shell around it, similar to the parachute structure reported by Jung et al. [18,19]. In their work, polymerization was conducted in the bilayer and upon polymerization, there is a complete phase separation of the polymer forming a blob on one side and leaving the other part of the vesicle completely free from polymer.

From the point of view of “control” over polymerization or RAFT control, it is very clear that Exp 6 is rather successful in achieving the desired structures. Exp 7, however, which has poor molecular control, produces undesirable morphology although pro-trusions might be a pre-cursor to nanobottle synthesis [15].

##### Polymerization of (DODAB-**Oligo 1**) with MA or MMA with Crosslinker (Exp 8–12)

To build up molecular weight quickly under starved condition and to fixate the polymer that is formed on the vesicle surface, a cross linker is introduced as the additional monomer in the nanocapsules synthesis. The type of crosslinker utilized is of the same family as the main monomer, acrylate or methacrylate.

**Figure 12:**
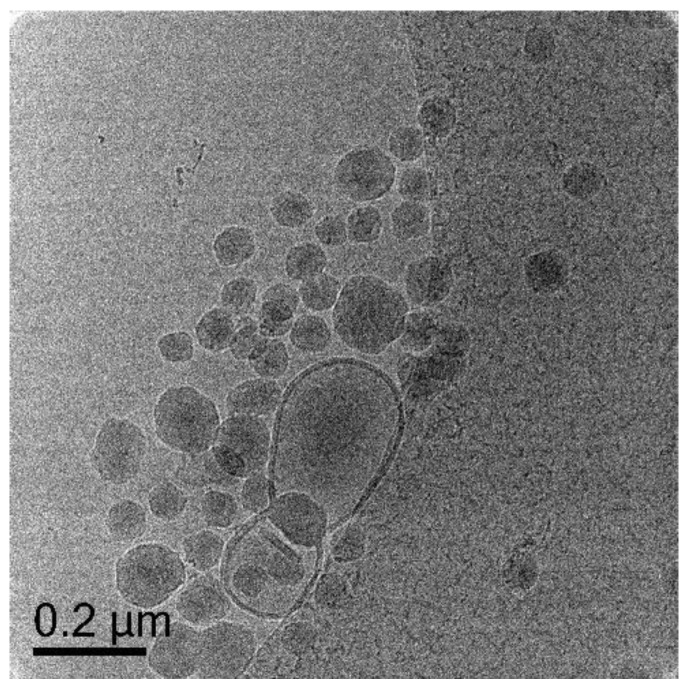
Cryo-TEM micrograph of Exp 8, polymerization of (DODAB-**Oligo 1**) with MMA/EGDMA. Additional images in Figure S7.

In Exp 8, MMA and ethylene glycol dimethacrylate (EGDMA) were used as the monomers. In this uncontrolled system, the presence of crosslinker results in the same particle morphology (Figure 12, Figure S7) as in Exp 7 in which pro-trusion and solid particles are observed (Figure 11B). This result further strengthens the earlier hypothesis on the criteria for successful nanocapsules synthesis via RAFT-mediated vesicles templated polymerization (monomer and its RAFT control).

**Figure 13:**
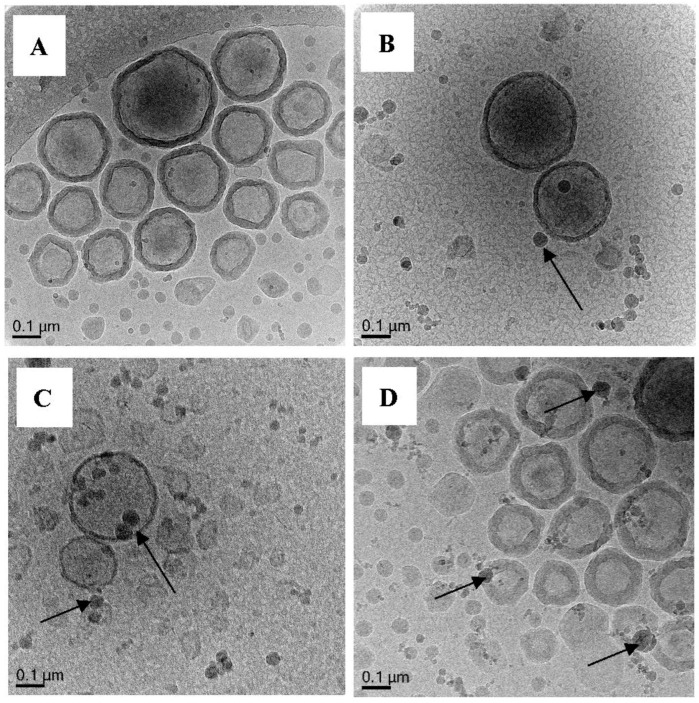
Cryo-TEM micrographs of Exp 9 (**A**), Exp 10 (**B**), Exp 11 (**C**) and Exp 12 (**D**). Polymerization of (DODAB-**Oligo 1**) with varying amount MA/EGDA. Some intense darks solids are crystalline ice particles (marked with arrows) introduced by contamination during sample vitrification. Additional images in Figure S8 (Exp 9) and Figure S9 (Exp 12).

In Exp 9–12, MA and EGDA were used in varying amounts. All of these experiments result in formation of nice hollow nanocapsules. When 0.5 mmol of EGDA is used (Exp 9, Figure 13A and Figure S8), most of the particles appear as hollow nanocapsules with a clear polymer shell of thickness of ca. 25 nm. When the amount of crosslinker is doubled (Exp 10, Figure 13B), the hollow nanocapsules appear to have thinner shells. However, when the crosslinker is 5× more (Exp 11, Figure 13C), the polymer shell appears irregularly shaped with uneven (not smooth) surface which is sort of typical morphology for highly cross-linked nanostructures. Exp 12 (Figure 13D, Figure S9) contains the same MA/EGDA ratio as Exp 9 but the total amount monomer fed is doubled. The polymer shell of the nanocapsules appears to be thicker with thickness of ca. 40 nm. These results confirm our hypothesis on the criteria of nanocapsules synthesis via RAFT-mediated vesicles template polymerization in which a crosslinker of the same family as the main monomer has to be added to fixate the polymer on the template during and after polymerization. Without this fixation through crosslinking, the polymer chain can easily detach from the vesicle surface resulting in a low amount of nanocapsules formed (Exp 6, Figure 11A).

We are confident from many cryo-TEM images that the nanocapsules are the majority of the particles in this synthesis techniques (Exp 9–12). A small population of solid and pro-trusion particles is still observed in the system. Several factors that can cause this are, among others:

- **macro-RAFT co-oligomer**. The free co-oligomer in the aqueous phase can chain extend in the presence of monomer and initiator. The co-oligomer is added in excess to make sure that there is enough co-oligomer to adsorb to the vesicle surface and also to provide electrostatic charge to stabilize the particles. The chemical composition distribution of the co-oligomer, however, is quite broad from the MALDI-ToF analysis and surface active species could be there.
- **DODAB vesicles**. It is well known that vesicular structures are very delicate [16,17,18], when subjected to high shear, ionic strength and temperature, they can break and disintegrate in small micelle-like particles or flattened vesicles.
- **Polymerization technique**. Even with slow monomer addition a diffusion into the vesicle bilayer might occur, that can lead to polymerization and phase separation into a pro-trusion morphology.
- **Cross-linker**. Addition of small amount of crosslinker (0.5–1 mmol) is enough to fixate and immobilize the polymer chain on the vesicle surface.

##### Polymerization of (DODAB-**Oligo 1**) with MA/BA and BA-(EGDA) (Exp 13–15)

With the successful synthesis of nanocapsules using MA combined with EGDA as crosslinker, we investigated the effect of butyl acrylate (BA) as the monomer. Based on the result of the solution polymerization, a good control should be achieved for RAFT polymerization of BA.

**Figure 14:**
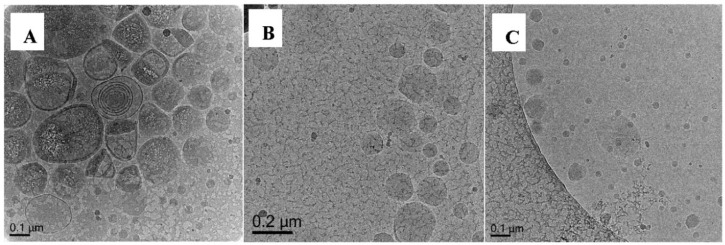
Cryo-TEM micrographs of Exp 13 (**A**), polymerization of (DODAB-**Oligo 1**) with MA/BA (10/1 wt); Exp 14 (**B**), polymerization of (DODAB-**Oligo 1**) with BA and Exp 15 (**C**), polymerization of (DODAB-**Oligo 1**) with BA/EGDA. Additional images in Figure S10 (Exp 13).

When mixed monomer of MA-BA in 10:1 by weight was used in the polymerization (Exp 13) we obtained a combination of morphologies we have seen so far i.e., capsules, pro-trusions, solids and additionally onions, capsules encapsulating another capsules (Figure 14A, Figure S10). This is a clear indication that BA, although present less than 10% in weight, can disrupt the nanocapsules formation. Polymerization of **Oligo 1** with pure BA (Exp 14, Figure 14B) and BA-EGDA (Exp 15, Figure 14C) do not result in nanocapsules at all. There are mainly solid particles of various sizes and no indication of nanocapsules even when polymerized with crosslinker (EGDA). A reason might be that BA is swelling the vesicle bilayer more strongly than MA (MA is more water soluble than BA) [28] and changing the vesicle structure. Another reason could be that the BA chain extended charged macro-RAFT co-oligomer is able to disrupt the vesicle structure. This could be similar to disruption of liposomes by amphiphilic random and block copolymers [29]. It was shown that the structure of the amphiphilic copolymer has an effect on which type of membranes are disrupted. Our amphiphilic macro-RAFT co-oligomer, chain extended with BA units resembles a block copolymer structure and might be more effective in disrupting the DODAB vesicles. The structure that is formed from chain extension with MA, where the MA block is more hydrophilic [28], might be less effective in disrupting the vesicle structure and therefore leading to successful nanocapsule formation.

#### 3.5.7. Polymerization of **Oligo 2A** with DODAB Vesicle (Exp 16-19)

To verify if the nanocapsules synthesis is RAFT agent dependent, another macro-RAFT co-oligomer of BA and AA (**Oligo 2A**) is synthesized from RAFT agent (BM1361) in 1,4-dioxane. Table 4 summarizes the results for the nanocapsules synthesis using **Oligo 2A**.

**Figure 15:**
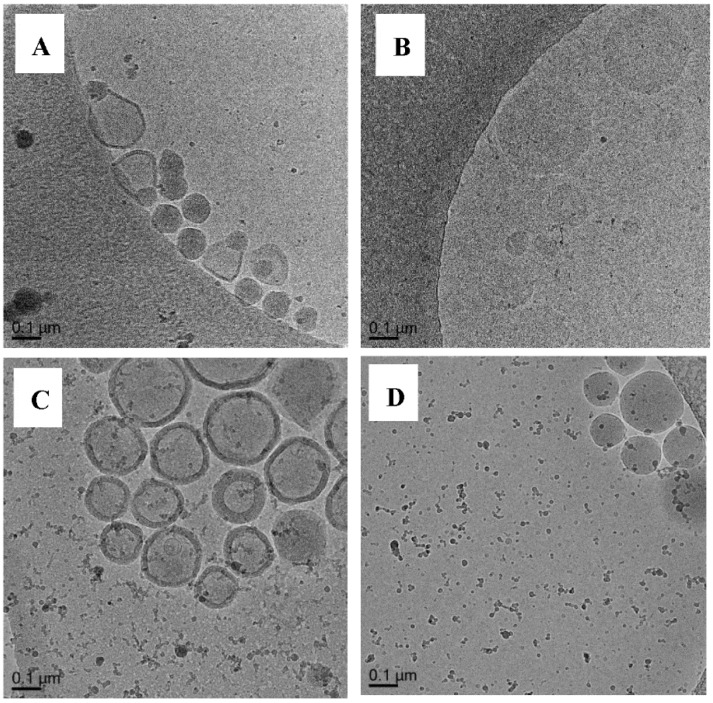
Cryo-TEM micrograph of Exp 16 (**A**), polymerization of (DODAB-**Oligo 2A**) with MMA; Exp 17 (**B**), polymerization of (DODAB-**Oligo 2A**) with MA; Exp 18 (**C**), polymerization of (DODAB-**Oligo 2A**) with MA/EGDA and Exp 19 (**D**), polymerization of (DODAB**-Oligo 2A**) with BA/EGDA. Additional images in Figure S11 (Exp 17) and Figure S12 (Exp 18).

Similar to **Oligo 1**, in uncontrolled system with MMA (Exp 16), the resultant particles are pro-trusion and solids (Figure 15A). When MA is polymerized without crosslinker (Exp 17), the particles do not seem to have a clear polymer shell and appear flattened/film like (Figure 15B, Figure S11). In the presence of EGDA as crosslinker (Exp 18), polymerization results in spherical capsules (Figure 15C, Figure S12) which is in agreement with results from **Oligo 1** (Figure 13). Finally, as expected from BA-EGDA (Exp 19, Figure 15D), no nanocapsule is observed and again the dominating morphology is that of solid particles.

The results of **Oligo 1** and **Oligo 2A** shows that the nanocapsules synthesis is not really RAFT agent dependent but this doesn’t mean that any RAFT agent will be suitable. The selected RAFT agent must be able to control the polymerization of the monomer. Initial selection of RAFT agent can be done by checking the RAFT agent—monomer compatibility in solution polymerization. This information is usually available from the RAFT agent supplier for major monomer classes. However, it is still best practice to determine the kinetics of RAFT co-polymerization in solution when synthesizing the oligomer. 

## 4. Conclusions

Successful nanocapsule synthesis via vesicle templated RAFT-based emulsion polymerization relies on:

1. starting with a stable and relatively mono-disperse vesicle dispersion
2. adsorption of oppositely charged macro-RAFT co-oligomer and RAFT controlled chain extension with a monomer that is slightly water soluble (MA works and BA does not work)
3. having some crosslinking to retain the non-equilibrium morphology
4. a good compatibility between RAFT agent and monomer

The nanocapsule is formed by growing layer of polymer on the outside of the stable vesicle by chain extending an oppositely charged RAFT oligomer under molecular control. Some of the earlier work did not use the correct combination of RAFT agent and monomer. Furthermore, it turns out that avoiding swelling of the vesicle bilayer by adding the monomers slowly is essential, as well as avoiding too hydrophobic monomers that can easily swell the bilayer and/or chain extend to amphiphilic copolymers that can disrupt the vesicle bilayer, leading to undesired morphologies.

With this thoroughly verified approach we now can synthesize nanocapsules in a cost-effective way, utilizing a simple emulsion polymerization like process. Post-loading of these nanocapsules with actives and selecting responsive polymers for the wall material opens up applications in areas like personal and consumer care, oil field chemicals application and biomedical applications.

## Figures and Tables

**Table 1:** Characterization of co-oligomer with NMR and GPC.

Co-Oligomer	M_n_, _theoretical_ (g/mol)	M_n_, _NMR_ (g/mol)	M_n_, _GPC_ (g/mol)	PDI, _GPC_
**Oligo 1** BM1481 (BA_6_-*co*-AA_9_) ^	1629	1631	2259	1.26
**Oligo 2A** BM1361 (BA_6_-*co*-AA_9_) ^	1708	1820	2244	1.28
**Oligo 2B** BM1361 (BA_6_-*co*-AA_9_) *	1708	1372	1920	1.31
**Oligo 2C** BM1361 (BA_6_-*co*-AA_9_) ^#^	1708	1421	1870	1.35
**Oligo 2D** BM1361 (BA_6_-*co*-AA_9_) ′	1708	1510	1953	1.32
**Oligo 3** (BA_4_-*co*-AA_8_)^^^	1418	1090	1241	1.74

^ **Oligo 1, 2A, 3** synthesized in 1,4-dioxane, * **Oligo 2B** in isopropanol, ^#^ **Oligo 2C** in ethanol and ′ **Oligo 2D** in bulk.

**Table 2:** Recipe for fortified vesicles for use in templated polymerizations.

(RAFT) Oligomer (10 mM, pH 7)	Volume (mL)			Charge Ratio Parameter (ξ)	[Oligo]/[V-501]
	RAFT Oligomer Solution	DI Water	DODAB (10 mM)		
**Oligo 1** [BM1481 (BA_6_-*co*-AA_9_)	50	50	100	4.5	2
**Oligo 2A** [BM1361 (BA_6_-*co*-AA_9_)	33	67	100	3.0	2
**Oligo 3** (BA_4_-*co*-AA_8_)	80	20	100	7.2	2

**Table 3:** Vesicles polymerization with **Oligo 1** [RAFT1481 (BA_6_-*co*-AA_9_).

Exp	Oligomer (10 mM)	DODAB Vesicles (10 mM)	Pre-polymerization Stability (Adsorption)	RAFT Control	Monomer	Cross-Linker	Post-polymerization Stability	Cryo-TEM (Particles Morphologies)
1.	**Oligo 1**	**✓** (unextruded)	**✕**	-	-	-	-	-
2.	**Oligo 1**	**✕**	**✓**	**✓**	MA (11.6 mmol)	EGDA (0.59 mmol)	**✓**	Solids (10–20 nm)
3.	**✕**	**✓**	**✓**	-	MA (11.6 mmol)	EGDA (0.59 mmol)	**✕**	-
4.	**Oligo 3**	**✕**	**✓**	-	MA (11.6 mmol)	EGDA (0.59 mmol)	**✓**	Solids (30–70 nm)
5.	**Oligo 3**	**✓**	**✓**	-	MA (11.6 mmol)	EGDA (0.59 mmol)	**✓**	Solids (100–150 nm), vesicles
6.	**Oligo 1**	**✓**	**✓**	**✓**	MA (11.6 mmol)	-	**✓**	Capsules, solids
7.	**Oligo 1**	**✓**	**✓**	**✕**	MMA (10 mmol)	-	**✓**	Pro-trusion, vesicles, solids
8.	**Oligo 1**	**✓**	**✓**	**✕**	MMA (10 mmol)	EGDMA (0.50 mmol)	**✓**	Pro-trusion, solids
9.	**Oligo 1**	**✓**	**✓**	**✓**	MA (11.6 mmol)	EGDA (0.59 mmol)	**✓**	Capsules
10.	**Oligo 1**	**✓**	**✓**	**✓**	MA (11.6 mmol)	EGDA (1.17 mmol)	**✓**	Capsules
11.	**Oligo 1**	**✓**	**✓**	**✓**	MA (11.6 mmol)	EGDA (2.94 mmol)	**✓**	Capsules
12.	**Oligo 1**	**✓**	**✓**	**✓**	MA (23.2 mmol)	EGDA (1.18 mmol)	**✓**	Capsules (thicker shells)
13.	**Oligo 1**	**✓**	**✓**	**✓**	MA/BA (11.6/0.78 mmol)	-	**✓**	Pro-trusion, solids
14.	**Oligo 1**	**✓**	**✓**	**✓**	BA (7.8 mmol)	-	**✓**	Flattened, film-like
15.	**Oligo 1**	**✓**	**✓**	**✓**	BA (7.8 mmol)	EGDA (0.59 mmol)	**✓**	Flattened, film-like

**Table 4:** Vesicles polymerization with **Oligo 2A** [RAFT1361 (BA_6_-*co*-AA_9_).

Exp	Oligomer (10 mM)	DODAB Vesicles (10 mM)	Pre-Polymerization Stability (Adsorption)	RAFT Control	Monomer	Cross-Linker	Post-Polymerization Stability	Cryo-TEM (Particles Morphologies)
16.	**Oligo 2A**	**✓**	**✓**	**✕**	MMA (10 mmol)	-	**✓**	Pro-trusion, solids
17.	**Oligo 2A**	**✓**	**✓**	**✓**	MA (11.6 mmol)	-	**✓**	Film-like particles, no clear shells
18.	**Oligo 2A**	**✓**	**✓**	**✓**	MA (11.6 mmol)	EGDA (0.59 mmol)	**✓**	Capsules
19.	**Oligo 2A**	**✓**	**✓**	**✓**	BA (7.8 mmol)	EGDA (0.59 mmol)	**✓**	Solids, flattened, film-like particles

## Supplementary Materials

The following are available online at http://www.mdpi.com/2073-4360/10/7/774/s1: Figure S1: Composition of **Oligo 2D** synthesized in bulk (BA/AA) from MALDI-ToF MS analysis; Figure S2: Cryo-TEM micrograph of Exp 2; Figure S3: Cryo-TEM micrograph of Exp 4; Figure S4: Cryo-TEM micrograph of Exp 5; Figure S5: Cryo-TEM micrograph of Exp 6; Figure S6: Cryo-TEM micrograph of Exp 7; Figure S7: Cryo-TEM micrograph of Exp 8; Figure S8: Cryo-TEM micrograph of Exp 9; Figure S9: Cryo-TEM micrograph of Exp 12; Figure S10: Cryo-TEM micrograph of Exp 13; Figure S11: Cryo-TEM micrograph of Exp 17; Figure S12: Cryo-TEM micrograph of Exp 18.
