# Supplementary material for: A Roadmap towards Successful Nanocapsule Synthesis via Vesicle Templated RAFT-Based Emulsion Polymerization
Source: Polymers (Basel). 2018 Jul 15;10(7):774. doi: 10.3390/polym10070774 (PMC6403744; doi:10.3390/polym10070774)
Supplement: Supplementary file 1 [file polymers-10-00774-s001.pdf]

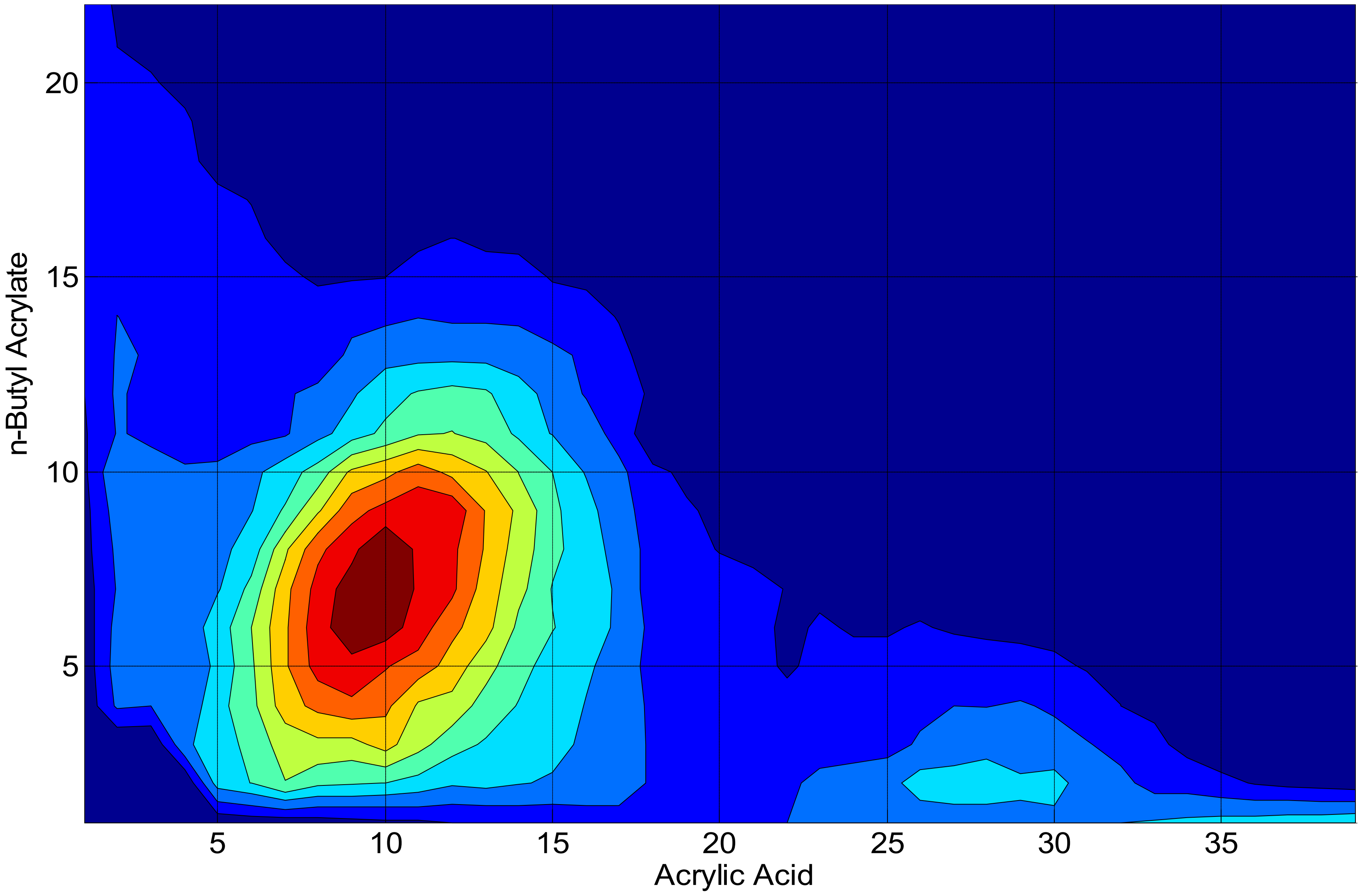

Figure S1. Composition of **Oligo 2D** synthesized in bulk (BA/AA) from MALDI-ToF MS analysis showing the number of acrylic acid units (x-axis) and butyl acrylate units (y-axis).

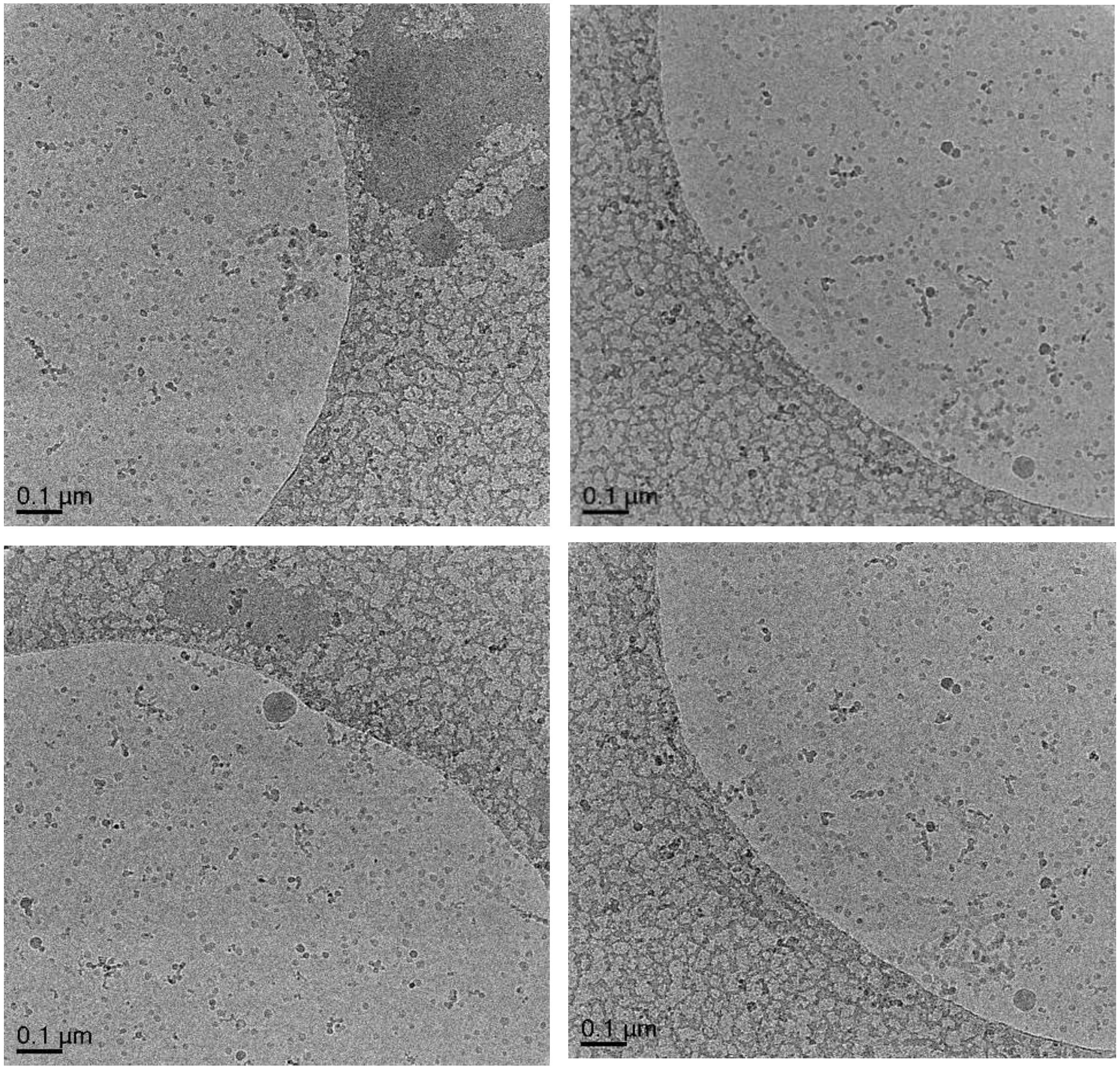

Figure S2. Cryo-TEM micrograph of Exp 2. Polymerization of MA/EGDA with **Oligo 1** without DODAB vesicle.

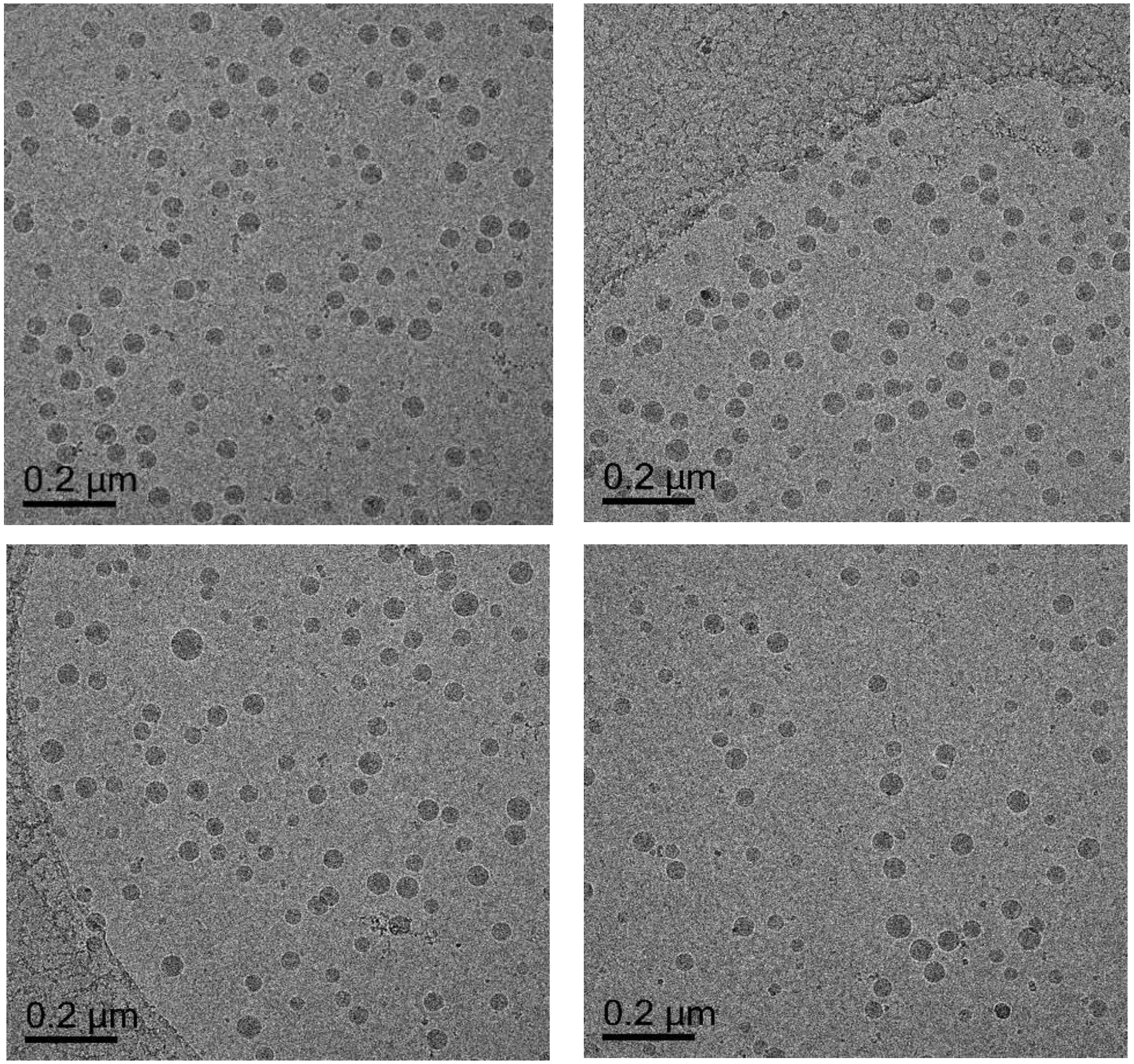

Figure S3. Cryo-TEM micrograph of Exp 4. Polymerization of MA/EGDA with **Oligo 3** without DODAB vesicle.

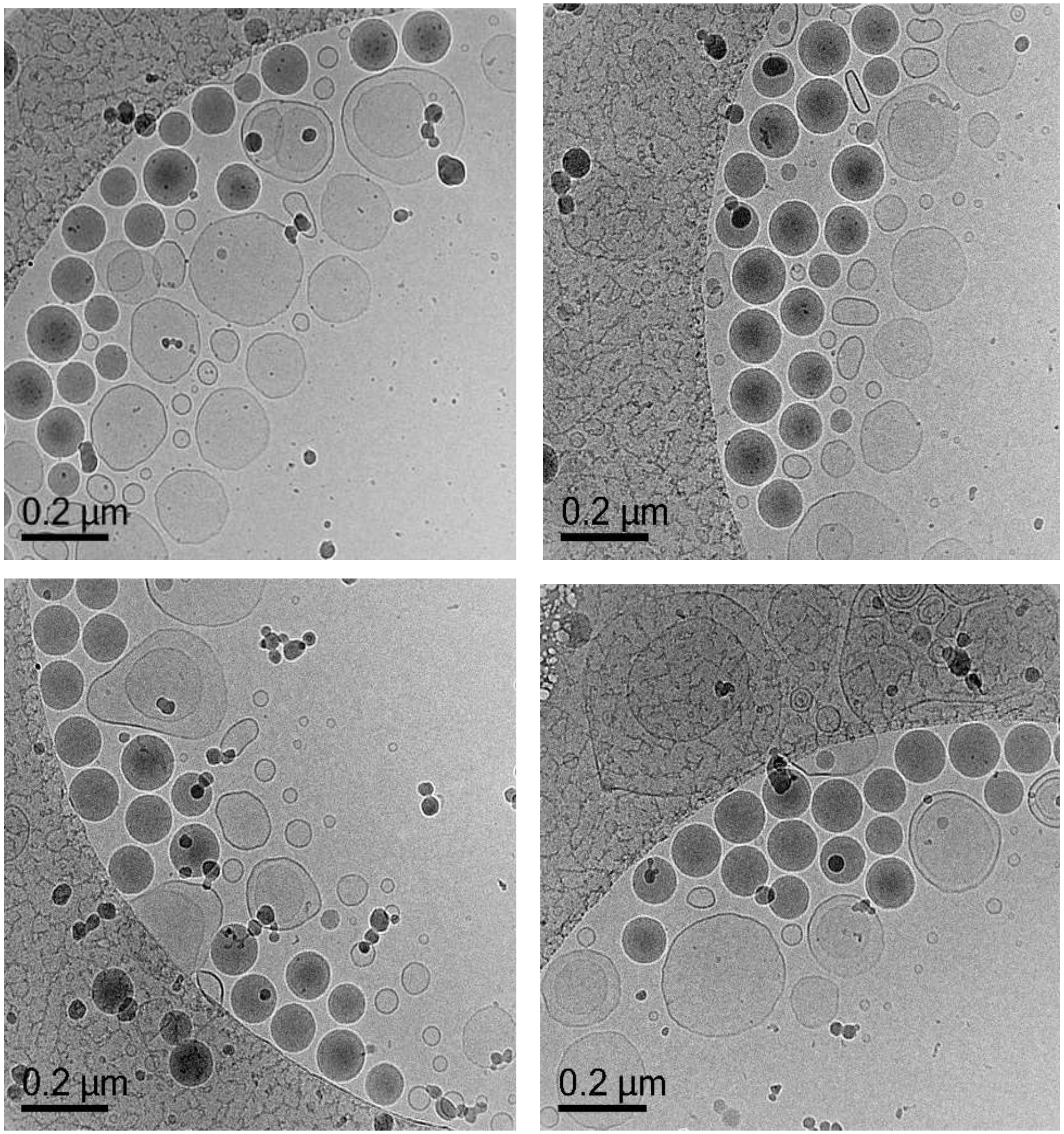

Figure S4. Cryo-TEM micrographs of Exp 5. Polymerization of MA/EGDA with **Oligo 3** and DODAB vesicle.

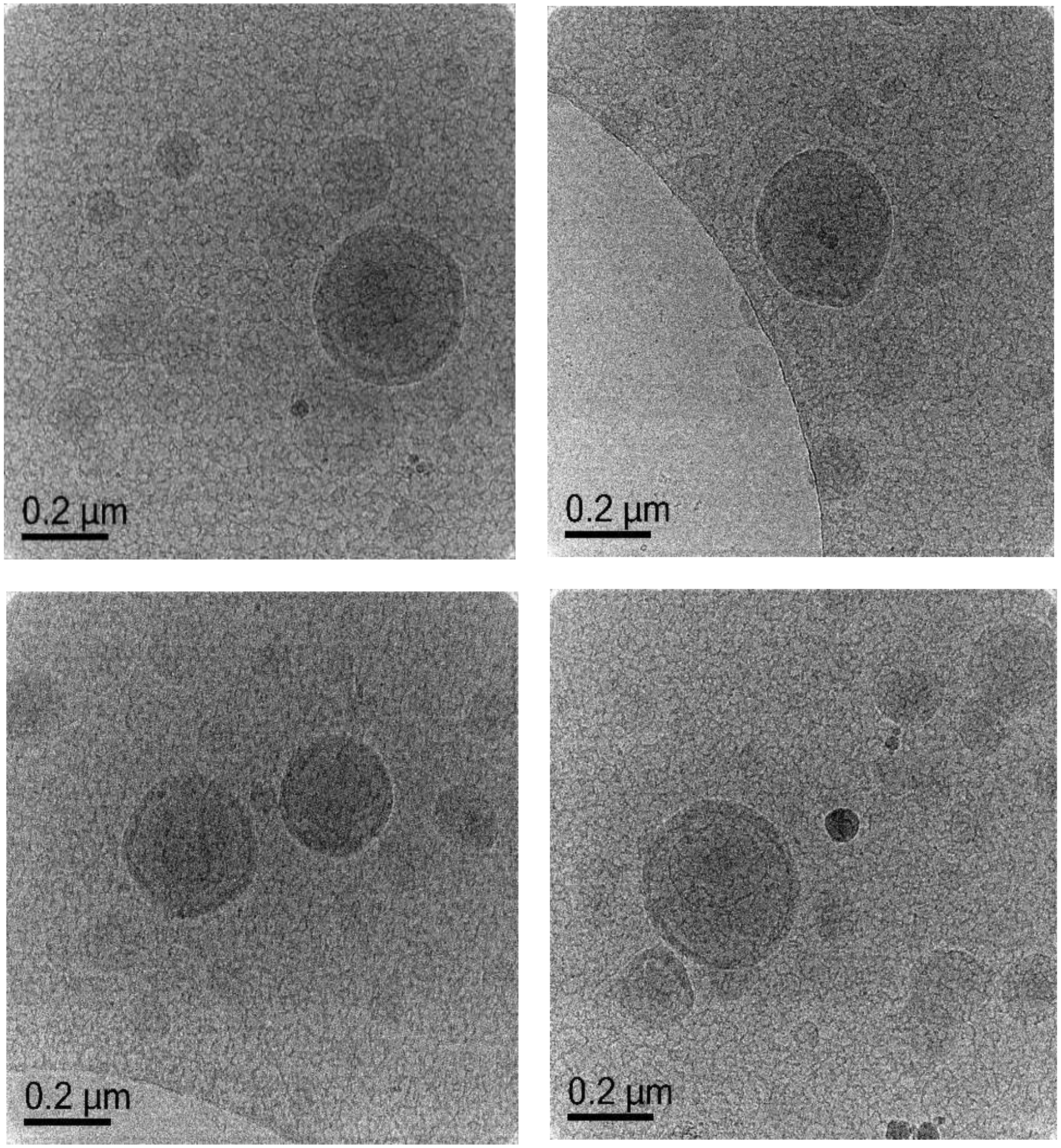

Figure S5. Cryo-TEM micrographs of Exp 6. Polymerization of DODAB-**Oligo 1** with MA.

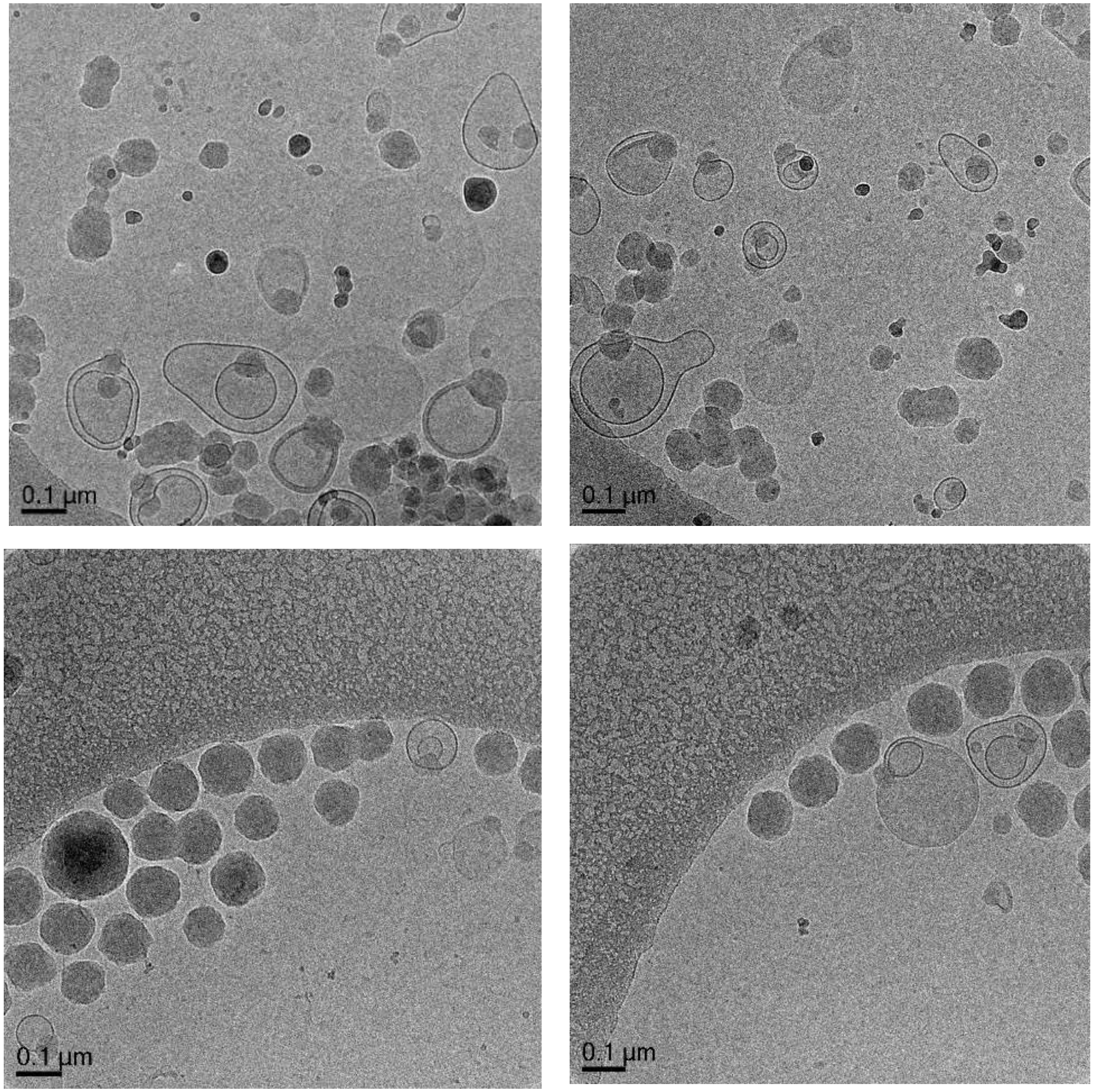

Figure S6. Cryo-TEM micrographs of Exp 7. Polymerization of DODAB-**Oligo 1** with MMA.

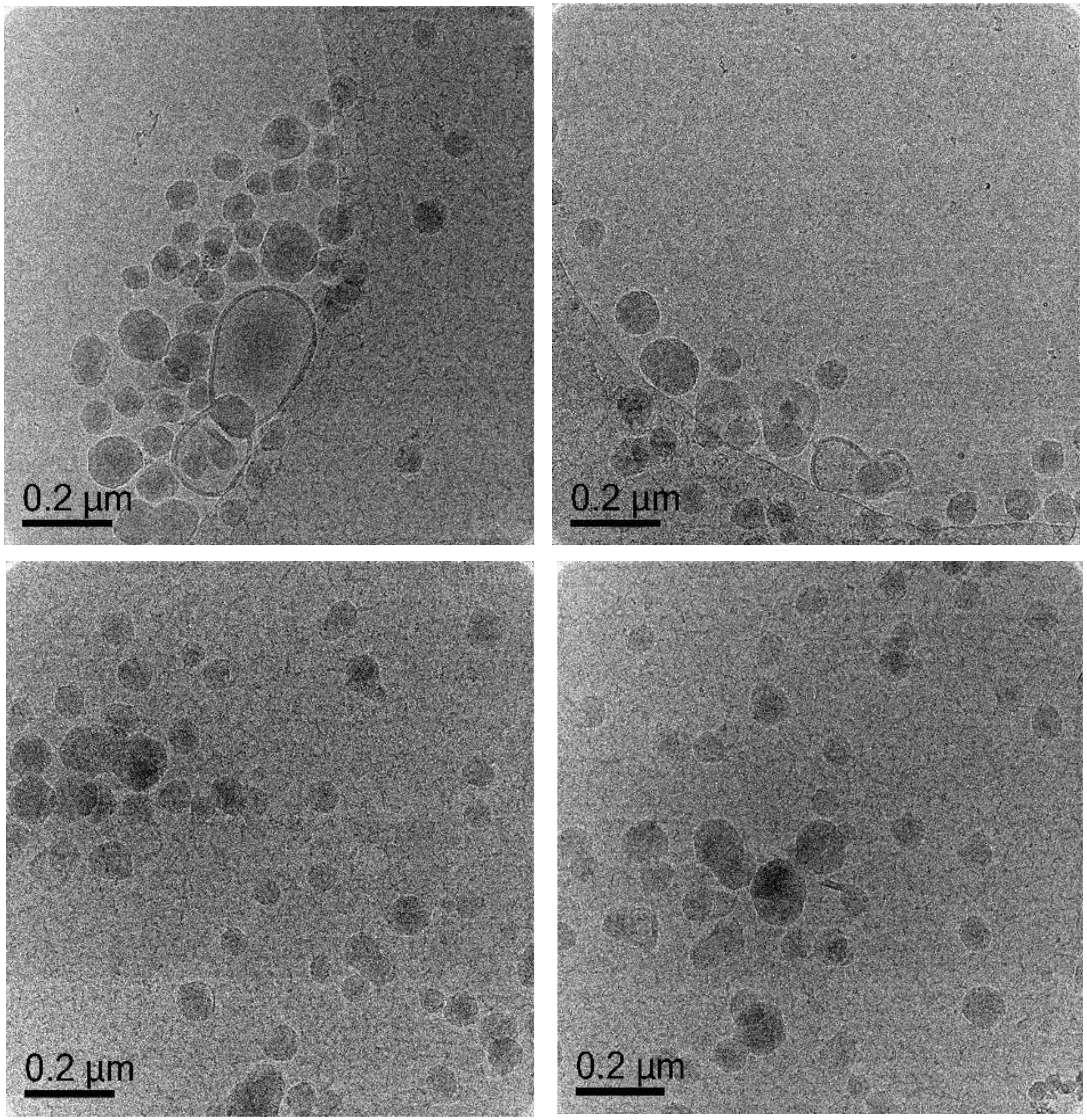

Figure S7. Cryo-TEM micrographs of Exp 8. Polymerization of (DODAB-**Oligo 1**) MMA/EGDMA.

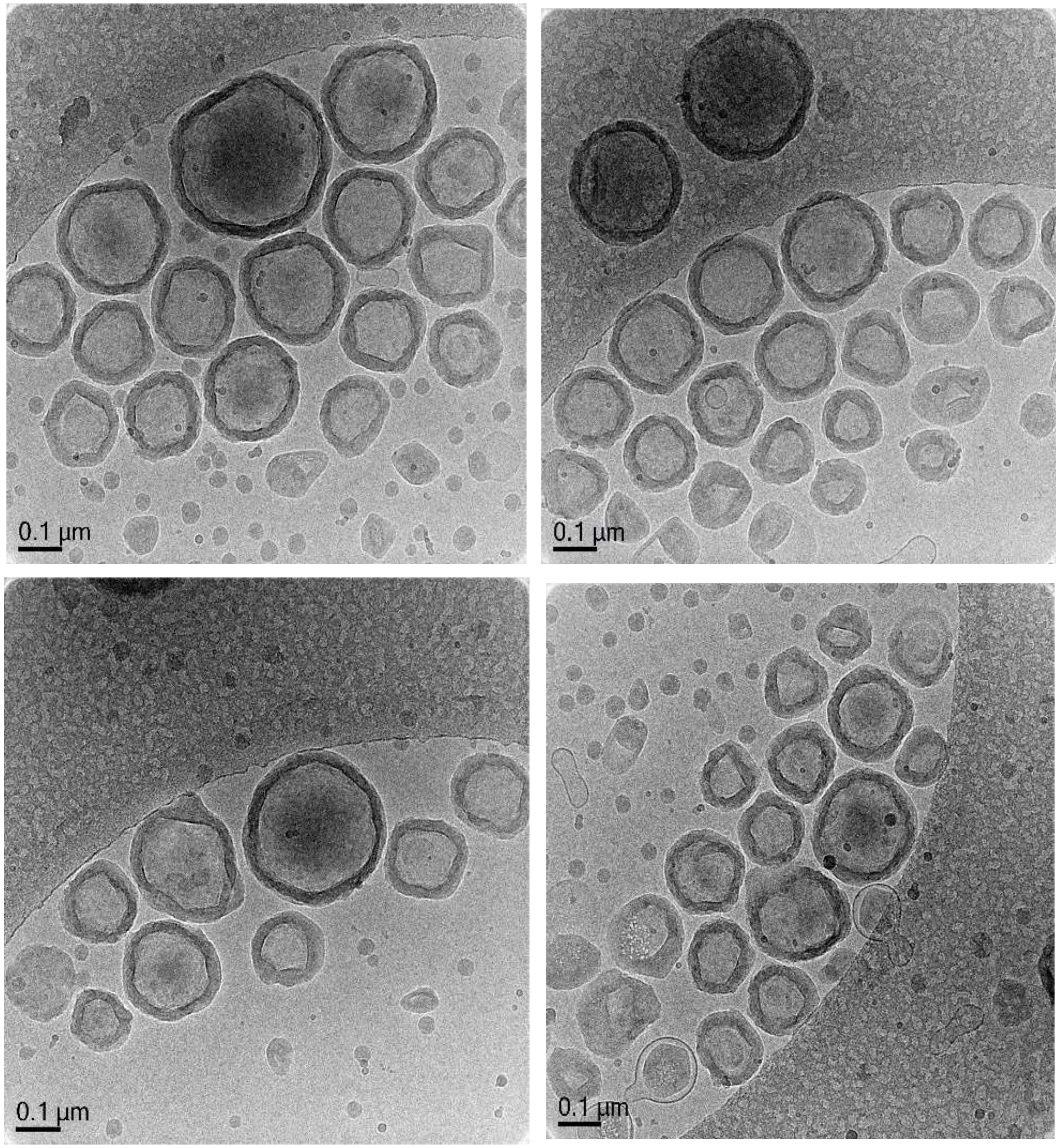

Figure S8. Cryo-TEM micrographs of Exp 9. Polymerization of (DODAB-**Oligo 1**) with MA/EGDA (11.6/0.59)mmol.

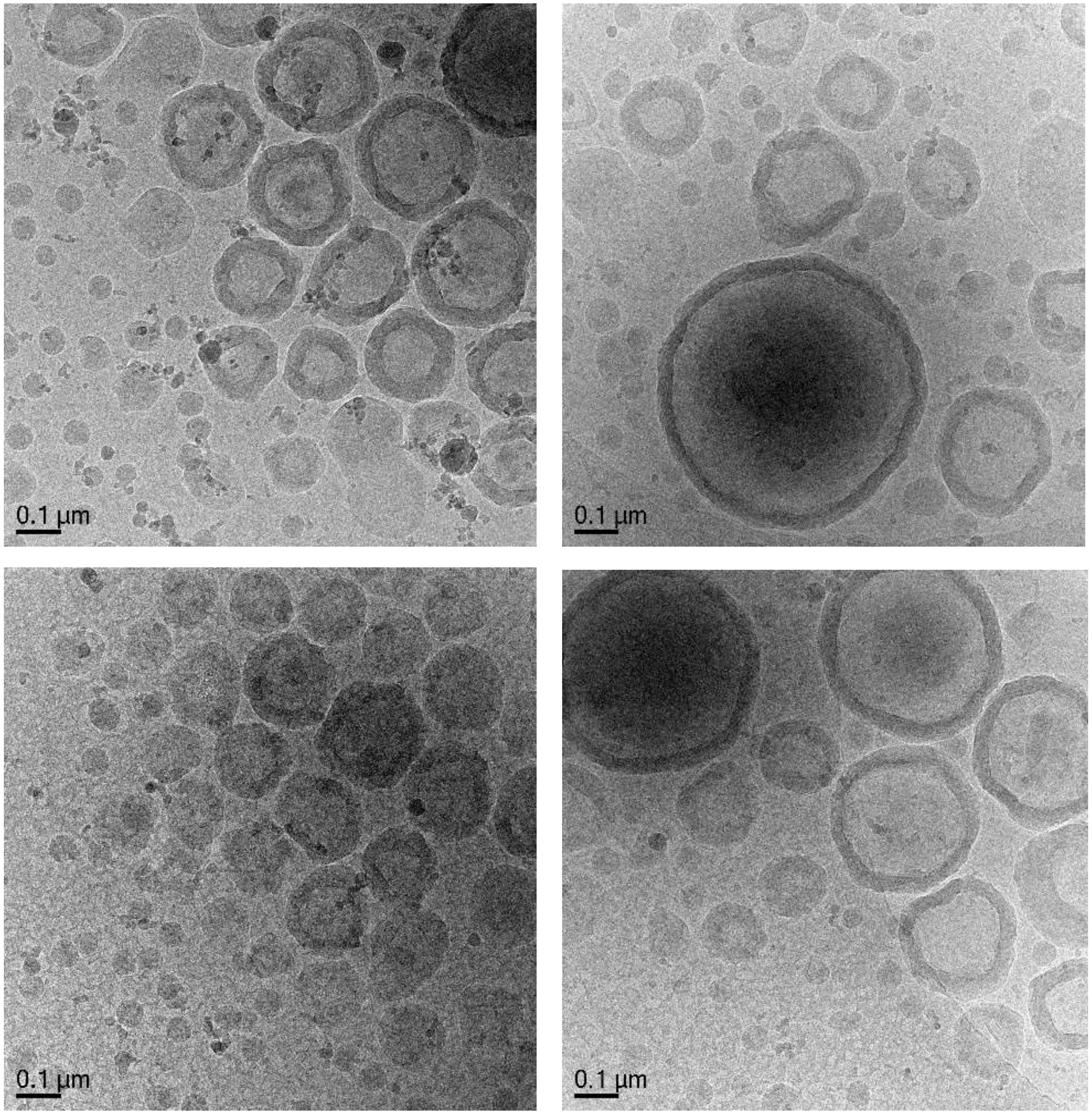

Figure S9. Cryo-TEM micrographs of Exp 12. Polymerization of (DODAB-**Oligo 1**) with MA/EGDA (23.2/1.18)mmol.

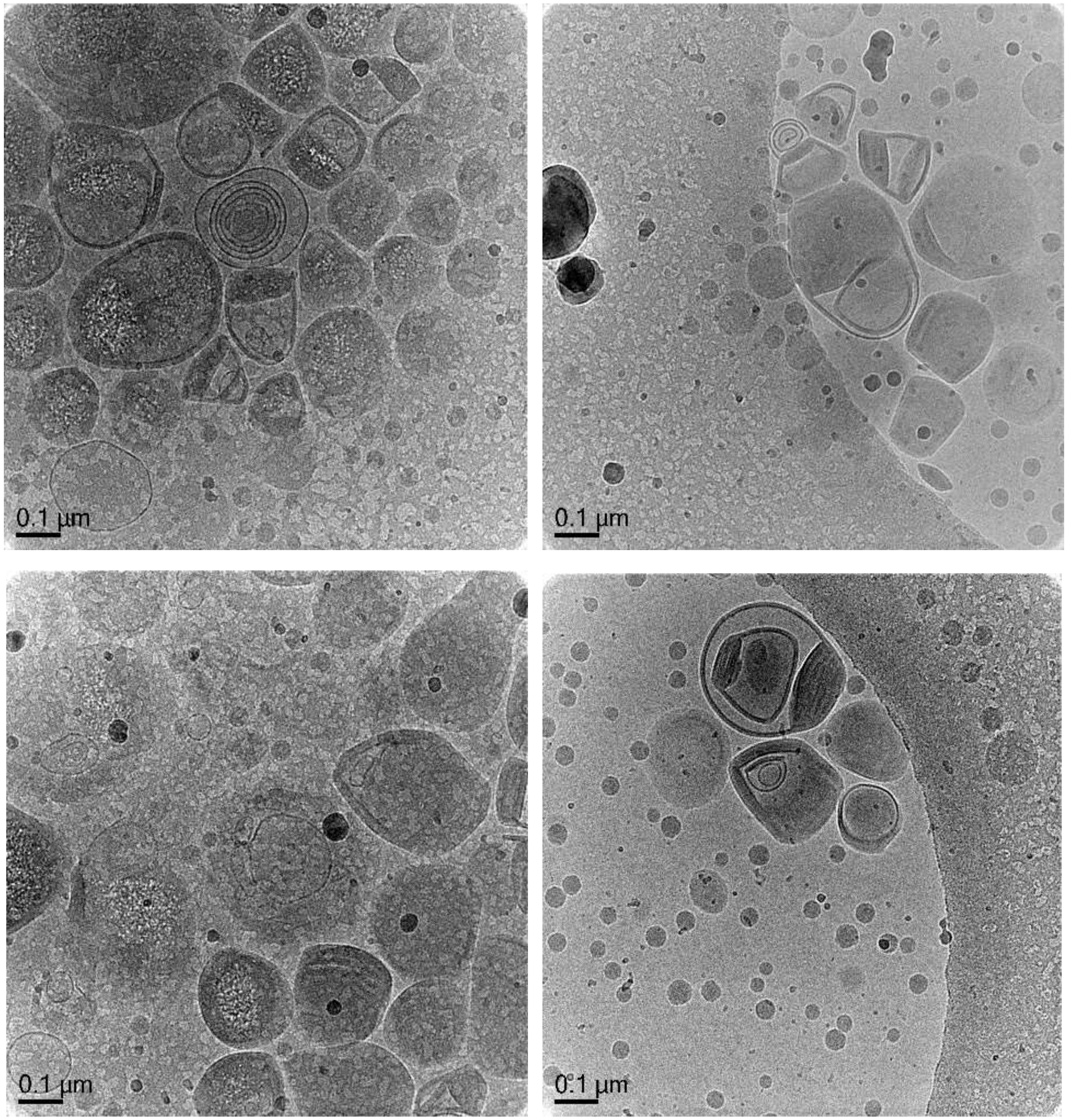

Figure S10. Cryo-TEM micrographs of Exp 13. Polymerization of (DODAB-Oligo 1) with MA/BA (10/1 wt).

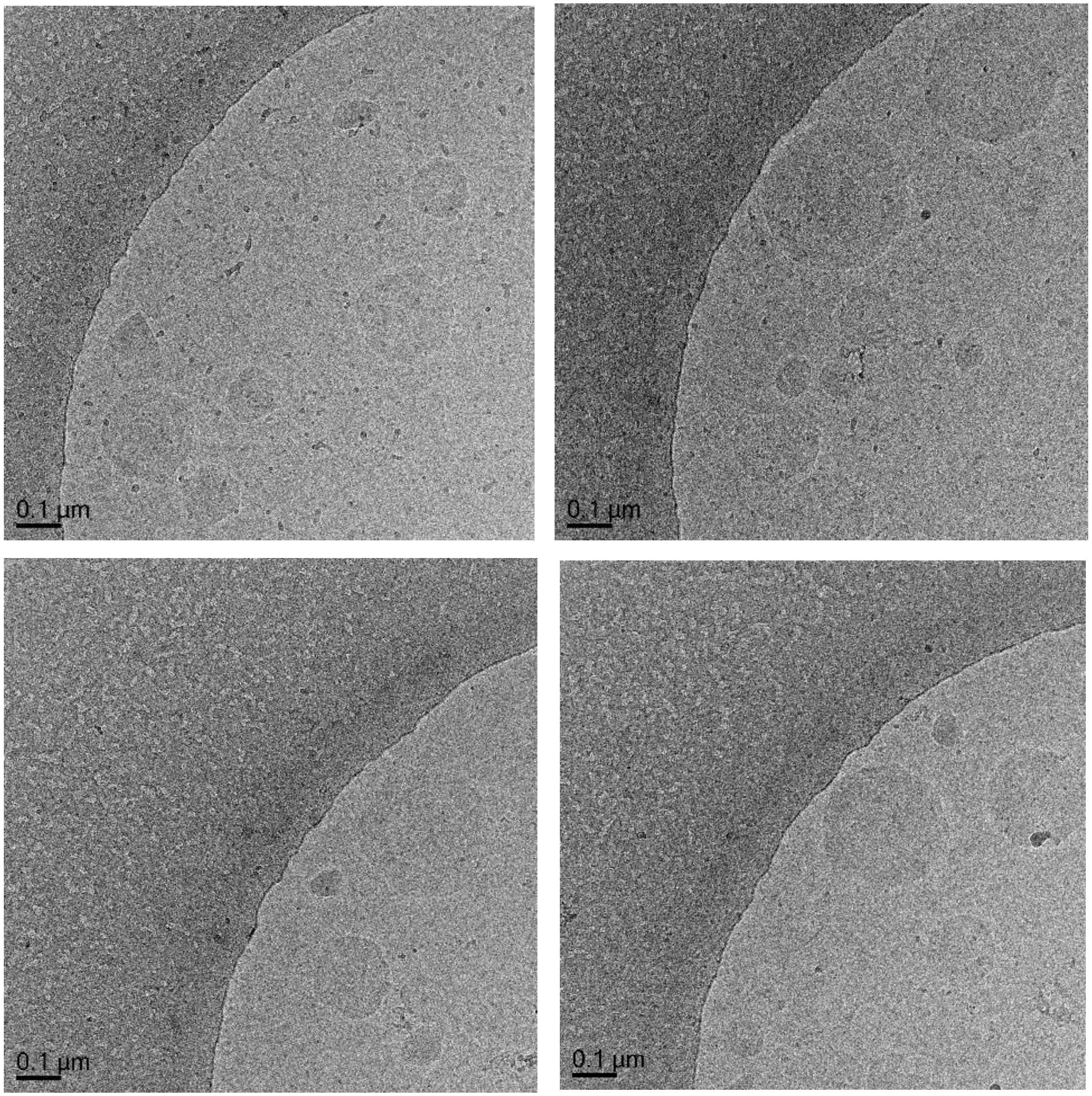

Figure S11. Cryo-TEM micrographs of Exp 17. Polymerization of (DODAB-Oligo 2A) with MA.

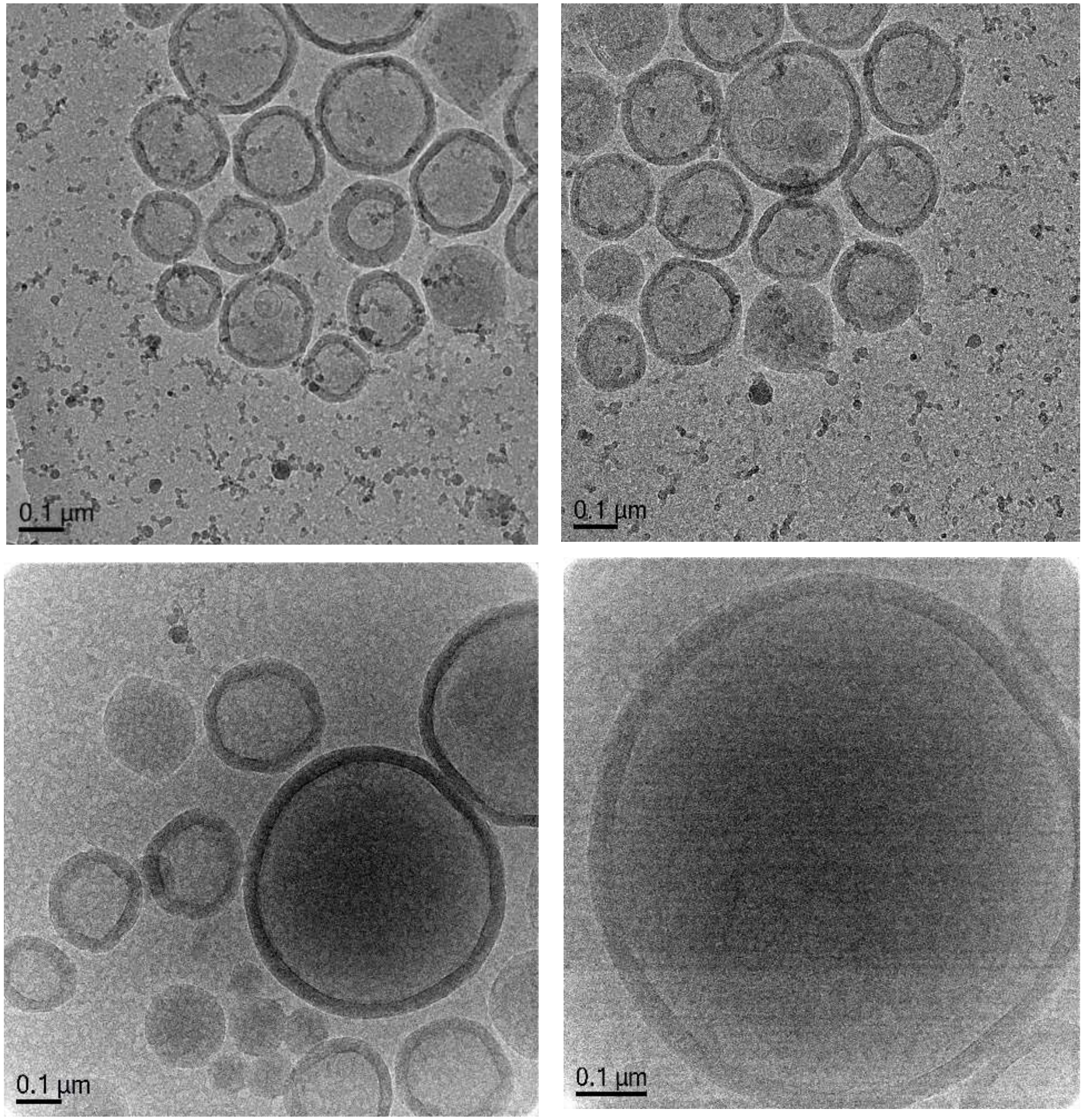

Figure S12. Cryo-TEM micrographs of Exp 18. Polymerization of (DODAB-Oligo 2A) with MA/EGDA.
